# Mapping Biodiversity and Setting Conservation Priorities for SE Queensland’s Rainforests Using DNA Barcoding

**DOI:** 10.1371/journal.pone.0122164

**Published:** 2015-03-24

**Authors:** Alison Shapcott, Paul I. Forster, Gordon P. Guymer, William J. F. McDonald, Daniel P. Faith, David Erickson, W. John Kress

**Affiliations:** 1 Genecology Research Center, Faculty of Science, Health, Education, and Engineering, University of the Sunshine Coast, Maroochydore DC, Queensland, Australia; 2 Queensland Herbarium, Queensland Department of Science, Information Technology, Innovation and the Arts, Brisbane Botanic Gardens, Toowong, Queensland, Australia; 3 Australian Museum, Sydney, Australia; 4 National Museum of Natural History, Smithsonian Institution Washington D.C., United States of America; The New York Botanical Garden, UNITED STATES

## Abstract

Australian rainforests have been fragmented due to past climatic changes and more recently landscape change as a result of clearing for agriculture and urban spread. The subtropical rainforests of South Eastern Queensland are significantly more fragmented than the tropical World Heritage listed northern rainforests and are subject to much greater human population pressures. The Australian rainforest flora is relatively taxonomically rich at the family level, but less so at the species level. Current methods to assess biodiversity based on species numbers fail to adequately capture this richness at higher taxonomic levels. We developed a DNA barcode library for the SE Queensland rainforest flora to support a methodology for biodiversity assessment that incorporates both taxonomic diversity and phylogenetic relationships. We placed our SE Queensland phylogeny based on a three marker DNA barcode within a larger international rainforest barcode library and used this to calculate phylogenetic diversity (PD). We compared phylo- diversity measures, species composition and richness and ecosystem diversity of the SE Queensland rainforest estate to identify which bio subregions contain the greatest rainforest biodiversity, subregion relationships and their level of protection. We identified areas of highest conservation priority. Diversity was not correlated with rainforest area in SE Queensland subregions but PD was correlated with both the percent of the subregion occupied by rainforest and the diversity of regional ecosystems (RE) present. The patterns of species diversity and phylogenetic diversity suggest a strong influence of historical biogeography. Some subregions contain significantly more PD than expected by chance, consistent with the concept of refugia, while others were significantly phylogenetically clustered, consistent with recent range expansions.

## Introduction

The international Convention on Biological Diversity (CBD) has highlighted the importance of conserving biodiversity in the light of climate change, changing environments and growing human populations [1]. Signatory nations have agreed to conserve 17% of areas with significant biodiversity by 2020 [1]. Given the financial and logistical constraints, this requires both efficient planning and identification of the most significant areas for biodiversity [2], [3], [4]. Rainforest and tropical ecosystems have been identified as particularly rich in biodiversity and have been targeted as of high importance in the REDD (Reducing Emissions from Deforestation and Forest Degradation) scheme [1]. This scheme relies on sound estimates of the geographic mapping and quantification of biodiversity. Australia is a signatory to the CBD and is one of the few developed countries with extensive and detailed GIS vegetation mapping that contains rainforest [www.environment.gov.au] [5].

Globally, Australian rainforests are relatively diverse at the family level and less so at the species level [6]. Historically, biodiversity assessments for conservation have been based on species richness, numbers of endangered and vulnerable species (IUCN) and levels of complementarity or endemism [3], [7]. This conventional approach has two limitations: Firstly, these biodiversity assessments ideally require a well-known taxonomy and extensive field surveys of biodiversity, and secondly, all species are treated equally in terms of their contribution to biodiversity [8], [9]. One promising strategy incorporates the phylogenetic diversity (PD), [10] measure into conservation planning. However, applications have been limited given the dependence on existing phylogenetic trees [10]. The use of DNA barcoding has the potential to help address these limitations, providing a more effective biodiversity assessment. For example, Costion et al. [11] have shown that DNA barcodes can be as effective as traditional field identifications in determining species in poorly known floras. A lot has been learned from PD assessments in absence of taxonomy in microbial ecology [12]. Further, such a strategy can provide measures of biodiversity that account for genetic differences among species. Critically, an approach using a standardised genetic measure means that unknown or unidentified species can be included in biodiversity assessments [13], [9].

The DNA barcode for an individual organism consists of the DNA sequence of a pre-determined part of the genome and enables a unique sequence identifier for each species [14], [15]. There has been considerable debate about the appropriate genes for use in DNA barcoding for plants, reflecting in part the great species diversity and smaller variation between species relative to animals [16], [17], [15]. It is now generally agreed that the plant barcode will consist of a minimum of two plastid genes, the conservative coding marker *rbcL* and more rapidly evolving region *matK*, with the intergenic spacer *trnH—psbA* recommended to enable greater discrimination [16], [17], [14], [13], [15]. The barcode sequence will enable a genetic distance between distant organisms and thus standardise measures of biodiversity. Further, DNA Barcodes have been used increasingly as a new tool, with applications in community ecology that have enhanced the understanding of the evolution and development of communities and has the potential to better estimate biodiversity [13], [14], [17], [18], [9] and perhaps provide more useful information about the causes underlying this variation.

There are many factors that contribute to levels of biodiversity and its distribution within the landscape [19], [20]. Island biogeography theory [21] and metapopulation dynamics [22] are key theoretical frameworks underpinning most ecological and conservation studies on species extinction [23], [24]. Fragmentation and metapopulation theories predict that greater connectivity will maintain higher diversity within habitat patches but lead to lower diversity between patches [22]. The species–area relationship (SAR) has been a cornerstone for conservation biogeography and conservation decision making [19]. Yet, there are surprisingly few studies that directly support this theory [25], [24], [20], [26], [27]. Gerstner et al. [28] found evidence that the relationship between species richness and sampled area differs considerably across the globe and is strongly dependent on the biome. However, Conservation rationale and assumptions are strongly influenced by these theories particularly in predictions of biodiversity loss as a result of habitat fragmentation and for estimation of extinction rates [29], [30]. Protected area reserve design has been highly influenced by the species area relationship [3], [31]. Many reserve design algorithums thus give priority to a set of well-connected reserves especially in light of climate change and the potential of organisms to migrate [3], [31], [10].

As part of its commitment to the CBD Australia has been mapped and classified into bioregions, which are broad-scale biogeographical units that distinguish areas with relatively coherent climate, geology, landform and biota (IBRA) (www.environment.gov.au), [32]. These are further classified and mapped according to subregions based on landform, geology and broad vegetation type [5]. The Australian National reserve system (NRS: www.environment.gov.au) aims to protect 10% of each bioregion; however, bioregions vary considerably in geographic extent, diversity and fragmentation. In Queensland a fine scale vegetation classification scheme has been developed (Regional Ecosystem, RE), based on vegetation structure and composition, substrate, geology and topography with grouping into bioregions [32] with the whole state mapped according to this scheme which is used as the basis for planning decisions [5]. Conservation planning is currently aimed at conserving representative examples of each of the identified vegetation types (RE) in order to best conserve the greatest representation of heterogeneity in biodiversity [33]. The ability to quantify biodiversity in an evolutionary framework, as enabled by phylogenetic reconstruction of communities from DNA barcode data, should greatly improve our ability to identify what bioregions, or fraction therein, will best conserve biodiversity.

In Australia, historical biogeography is thought to have significantly impacted the patterns of diversity within the landscape and on the phylogenetic composition [34], [35], [36]. Past climate change led to the contraction of rainforest to protected refugia followed by subsequent expansions with later fluctuating climatic cycles [37], [38], [34]. These impacts combined with older historic Gondwanaland patterns of connectivity, and expansions of tropical floras are thought to have shaped both the phylogenetic composition and patterns of diversity and endemism of Australian rainforest [39], [35], [36]. Kooyman et al. [40] found phylogenetic evenness (over-dispersion) more evident in north Queensland rainforest refugia, whereas phylogenetic clustering was more evident in southern Subtropical/temperate areas of northern NSW (New South Wales) due to recolonization from historically reduced species pools. Rainforest ecosystems are frequently typified by high levels of spatial heterogeneity. Some hypothesize that it is heterogeneity rather than area *per se* that promotes higher biodiversity [20]. Rainforests are generally thought to be found in habitats with high resource availability with these relatively benign environments thought to enable a higher diversity of species to co-exist [6], [41]. When environmental conditions become limiting, greater selection pressures and competition, result in lower diversity [37]. Thus where rainforest occurs across steep environmental gradients species diversity and composition is expected to vary [42], [43]. Several authors have found that rainforest with different environmental and evolutionary histories will differ in their phylogenetic diversity and patterns and that phylogenetic composition is often non-random [44], [14], [18]. Costion et al. [10] found that the presence of a relatively large share of immigrant Indo-Malayan lineages can increase the PD of Australian tropical rainforest areas relative to those with lesser shares.

**Fig 1:**
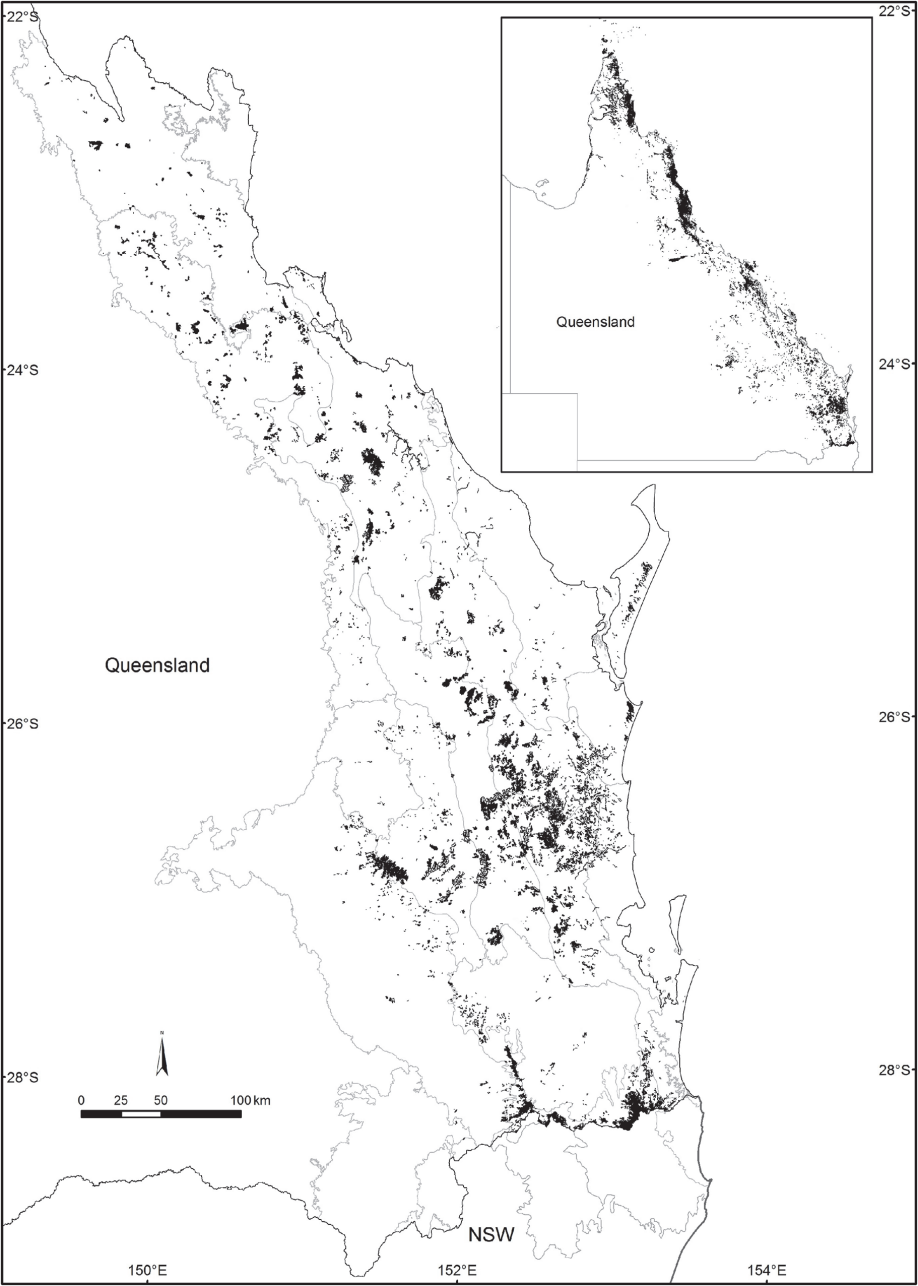
Map showing the distribution of rainforest in Queensland with the SE Queensland region expanded and the subregions indicated.

In Australia, rainforest crosses latitudinal gradients from tropical to temperate but is mostly located in Queensland within a narrow subcoastal band bounded by steeply declining rainfall gradients west of the Great Dividing Range (Fig. 1) [6], [45], [35]. Relictual Gondwanan, temperate and more recent tropical floristic elements extensively overlap particularly in the subtropical regions [37], [38], [35], [46], [36]. Variation in temperature, rainfall, geology, topography, soil nutrient availability and drainage has led to heterogeneity in rainforest structure and physignonomy [42], [45], [43], [35].

Australian rainforests contain high levels of biodiversity for the continent but have been reduced in size due to anthropogenic land modifications (Fig. 1) [6], [47].The tropical rainforest of North Queensland (N Qld) are less anthropogenically fragmented (mostly 70% or more remnant vegetation remaining) and better protected (Wet Tropics World Heritage Area) than the subtropical rainforest of South East Queensland (SE Qld) where significantly greater fragmentation has taken place due to agriculture and urbanisation and in some areas less than 10% of remnant vegetation remains (Fig. 1) [5].The fragmented nature of the protected areas remaining make the rainforests of SE Queensland more vulnerable to the impacts of climate change as there is less potential for species to move to track environmental change [48]. Thus identifying and prioritizing those areas that are most diverse and most endangered is of special importance in the SE Queensland bioregions and their associated subregions.

SE Queensland was selected for this study as it is a biogeographically coherent region that has been relatively poorly studied in terms of its rainforest diversity relative to the Wet Tropics rainforests and yet it is under higher pressure from weed incursions and urban expansion with a largely *ad hoc* approach to its conservation in the past. This study aimed to create a three marker DNA barcoded library of SE Queensland rainforest plants and to use these barcodes to enable a more sophisticated assessment of biodiversity for the extant SE Queensland rainforest estate that incorporates phylogenetic dissimilarity as a way to quantify evolutionarily scaled biodiversity. The study aimed to quantify rainforest biodiversity within SE Queensland bio subregions and compare levels among sub regions, as well as to contrast that with traditional methods for assessing rainforest biodiversity for SE Queensland bio subregions and to investigate the use of these results for rainforest biodiversity conservation.

## Materials and Methods

### Sampling

**Fig 2:**
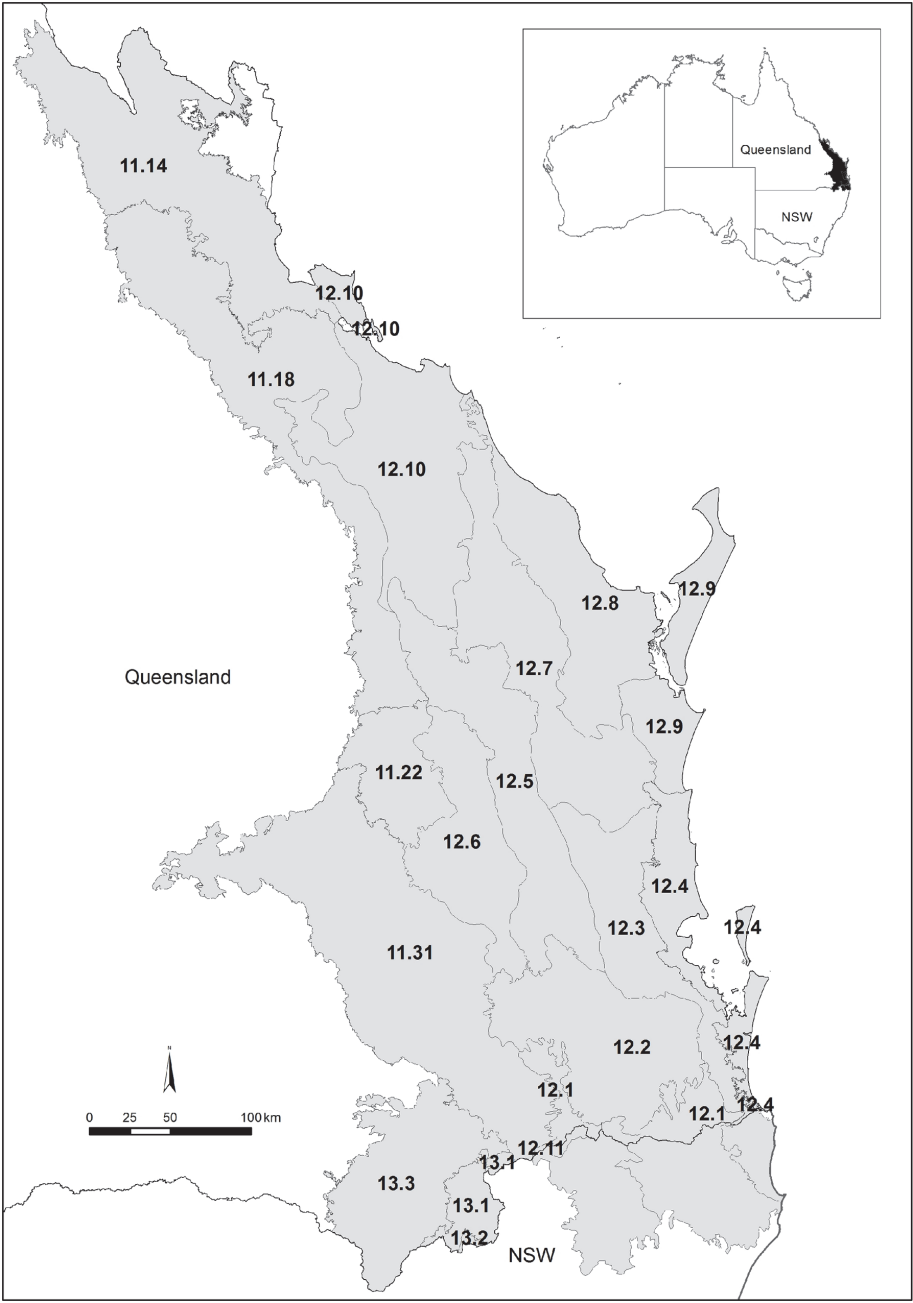
Map of Australia indicating the SE Queensland study region in solid back and the State boundaries identifying Queensland (Qld) and New South Wales (NSW). In the expanded box the SE Queensland study region is shown with the location and boundaries of the subregions marked and their codes indicated.

A complete list of SE Queensland rainforest vascular plants (Trees, shrubs vines and herbs excluding ferns and epiphytic orchids) was compiled from the Queensland Herbarium databases which include an extensive set of field survey data used for vegetation mapping in addition to the herbarium records. There are 61 rainforest species that are endemic to SE Queensland. The taxonomy is well known and the region relatively well surveyed compared to rainforest in other countries [49]. Epiphytic orchids were excluded due to difficulty in collection and we considered the project could be extended to include ferns at a later date but it was beyond the scope of the project at the time of collection. For the purposes of this study SE Queensland was defined as covering rainforest occurrence from the tropic of Capricorn (Rockhampton) to NSW including the Southeast Queensland Bioregion and several adjacent subregions from the Brigalow Belt and New England Tableland Bioregions [32] (Figs. 1 and 2). A ‘dry’ band of regional ecosystems (Brigalow Belt Bioregion) north of the Tropic of Capricorn extends right to the Pacific Ocean foreshore and the resultant reduction in rainforest occurrence and inherent diversity as a result of drier climatic conditions provide a strong biogeographic barrier. South from the Queensland border, the Burringbar—Conondale Ranges subregion of the Southeast Queensland bioregion extends into northeast NSW based on the extent of the Mt Warning caldera south to the Clarence River; south of this caldera and its associated volcanic flows, rainforest structure and diversity once again greatly decreases. As a result the area studied here forms a relatively coherent biogeographical unit with inferred barriers to continuity north and south due to less suitable climatic and substrate factors.

Botanists from the Queensland Herbarium, staff and students from the University of the Sunshine Coast, and local field botanists collected fresh material from the field with the aim to collect at least one specimen of each species from within the SE Queensland rainforest estate (Fig. 1). Specimens were thus collected from hundreds of sites across this distribution depending on their natural distribution. All collectors were provided with a set of protocols to ensure consistency in collection methods. The relevant Scientific purposes permits WISP10022311, WITK10022211 and TWB/29/2011were obtained from the Queensland Government Department of Environment and Resource Management which included permits to collect endangered and vulnerable species in addition a Research Activity permit was obtained from the Sunshine Coast Regional Council to enable collection on council land. Queensland Herbarium staff were covered to collect vouchers under their own departmental scientific permit as this is part of their core business. For each specimen both a herbarium specimen voucher including GPS location and a DNA specimen voucher preserved in silica gel were prepared. All herbarium vouchers and DNA specimens were submitted to the Queensland Herbarium (BRI) to be added to their collections and the identification of all specimens was confirmed by Herbarium staff and corrected where required. A duplicate DNA voucher was subsampled and lodged at the National Museum of Natural History (NMNH) within the Smithsonian Institution where the DNA barcoding analysis took place. The extracted DNA samples are held in the Kress collection at the United States National Herbarium at the Smithsonian Institution (US). Final collection consisted of 913 samples of 770 species from 111 families of which 120 were species collected from more than one location. The designation of species to families was consistent with the modification of the APGIII classification [50] as used by the Census of Queensland Flora [49].

### DNA Barcoding and Sequence alignment

Genomic DNA was extracted from silica dried tissue samples of all 913 samples following the methods of Ivanova et al. [51] as used by Kress et al. [13]. We undertook PCR and sequencing of all samples at three accepted DNA barcode markers, *rbcL*, *matK*, and the *psbA*-*trnH* intergenic spacer from the plastid genome following the methods outlined in Kress et al. [13]. Raw forward and reverse sequences were entered into the Geneious (Geneious 6.1.7) program where contigs were made and sequences for each sample at each marker were checked and edited to ensure quality and accuracy. The *rbcL* marker was aligned using ClustalW [52] implemented in Geneious. The *matK* marker was aligned using back translation through the program transAlign [53] followed by manual alignment in Geneious. The *psbA*-*trnH* sequences were sorted by family then aligned with MAFFT [54] as implemented by Geneious, then merged with the consensus aligner of MUSCLE [55] implemented in Geneious, into a single global alignment for *trnH*-*psbA*. This is an extension of the fully nested alignment employed by Kress et al. [13] which ameliorates the problem of model assignment to the different nested elements of the *trnH*-*psbA* regions by coaleseing them into a single aligned block to which a single model may be applied for purposes of phylogenetic reconstruction. The barcode data set was submitted to the BOLD database and sequences submitted to Genbank.

A preliminary tree was constructed for each marker to check for correct sample identification and sequence quality, whereupon samples that appeared significantly out of expected phylogenetic position were checked and corrected or removed from analysis. As a result of the preliminary checking some samples were re analysed for one or more loci to improve sequence quality. Given that this set of species consisted of many families with few representatives we decided that we would create an improved final phylogeny by using a large rainforest dataset order to better estimate species relationships and branch lengths for the SE Queensland rainforest species set. We aligned the SE Queensland data with a larger dataset derived from CTFS plots [56] creating a dataset of over 3000 species. The same three genes were used in the Queensland and CTFS datasets, and data from each gene (*rbcL*, *matK* and *psbA-trnH*) were aligned for all taxa, and then the three alignments were concatenated to make a three gene alignment containing all Queensland plus all CTFS taxa.

### Phylogenetic reconstruction

The aligned 3-marker matrix was uploaded to the CIPRES supercomputer portal [57] for phylogenetic reconstruction. We implemented RAxML-HP2 on XSEDE option in CIPRES portal [57], [58], [59] in conjunction with a constraint tree (S1 Output). The constraint tree was derived from Phylomatic [60] plants (http://phylodiversity.net/phylomatic/) using the R20120829 tree based on the APGIII base tree [50]. This tree was modified, in Mesquite [61] (http://mesquiteproject.org/mesquite/mesquite.html), where each order was collapsed to a polytomy. This enforced taxonomic relationships at the level of order and above, but allowed inference of relationships based on aligned nucleotide sequence below the level of order. We further employed data partitions that estimated a separate maximum-likelihood model for each marker (*rbcL*, *matK* and *trnH*-*psbA*). Thus we used one constraint tree for the entire phylogeny, but the constraint tree was collapsed such that each order was a polytomy and we let the sequence data sort out relationships within the orders. For the best tree search eight individual search replicates were initiated, each starting from random tree, to search for a best scoring likelihood tree we performed 96 maximum likelihood search replicates in searching for the most likely phylogeny. The 96 searches were done for both the phylogeny using a constraint, and for the phylogeny where no constraint was used evaluate the need for implementation of a constraint in a large phylogeny. The non-constrained phylogeny exhibited a number of polytomy at the base of the phylogeny, which we know from experience are not true and are a function of relatively little data used to infer those very deep relationships. Thus we did not employ the non-constrained phylogeny in our analysis and instead used the constrained phylogeny. The best tree, constructed with the APGIII derived constraint, with all 3000 taxa, was exported and opened in Geneious and rooted by the oldest taxon group in the tree (ferns). We then used the program PATHd8 to date the tree which estimates node ages by mean path lengths from the node to the leaves correcting for deviations from a molecular clock suggested by reference nodes [62]. Within this program the phylogeny was converted to a fully ultrametric chronogram by assigning the age of 9 Orders, using the same set of fossil dates as well as including a very old date for angiosperms of 250 mya employed in [63] in order not to greatly compress the branch lengths of the oldest nodes. A full list of fossil dates used in this dating procedure are given in S1 Dataset. A chronogram for the SE Queensland rainforest taxa was then pruned from this 3000 taxon ultrametric chronogram using the package APE in R [64]. Thus we used dating as a way of calibrating molecular branch lengths to generate an ultrametric phylogeny. This dated chronogram was then used in all subsequent analyses.

### Bioregion Subdivision

Each of the SE Queensland rainforest species used our final DNA barcode analysis was classified according to its membership in a subregion community [5], [32], using the databases of the Queensland Herbarium, resulting in a community species list for each subregion (Fig. 2). We used this list to calculate the species richness (SR), and number of families (Fam) represented in each subregion. We constructed a species accumulation curve using species composition of each subregion, starting with the most species rich subregion progressively adding the next species rich subregion until all species were accounted for. We recorded for each subregion its total area (Area), the area of rainforest that has been estimated prior to clearing (preclearing, Pre RF) and the area of remnant rainforest remaining (Rem RF) using data available [5]. Thus we were able to calculate the percentage of preclearing rainforest remaining (% Rem RF) and remnant (Rem RF%sub) and the percentage of the subregion area occupied by the original rainforest preclearing (Pre RF% Subregion), the percentage of the subregion area occupied by remnant rainforest (RemFR%sub) as well as the Regional Ecosystem (RE) vegetation types present in each subregion and percent of the subregion area under Protected Area (National Park, Conservation Reserve etc.) and forestry (e.g. State Forest) land tenure using the subregional analysis as of 2009 [5].

To assist in interpretation of the results, we visualised the SE Queensland rainforest species tree using the interactive Tree of Life (iTol) [65] software web site program. We presented the species composition within the tree of each subregion community by a subregion specific coloured bar and coded each subregion as concentric patterns of coloured bars around a circular representation of the tree. We used the SE Queensland chronogram and the subregion composition community files to calculate Phylogenetic Diversity (PD) [10], mean-phylogenetic-distance (MPD) and mean-nearest-taxon-distance (MNTD) [66] for each subregion and for the whole SE Queensland rainforest community, using the PICANTE package in R [67]. Standard effects sizes were calculated for MPD and MNTD under the “sample.pool” randomization model using 999 randomizations to test for significant differences among subregions compared to random giving us NRI and NTI measures following the protocols outlined in Swenson [68].

To further investigate diversity among subregions we undertook Spearman’s Rank Correlation analysis between Diversity metrics (SR,PD, MPD,MNTD, Fam) to confirm expected relationships among the diversity measures and the subregion rainforest parameters (Area, Pre RF, Rem RF, % Rem RF, Rem RF%sub, Pre RF% Subregion). We also plotted the log of subregion species richness against the log of subregion PD to further investigate their relationship and a standard linear regression line fitted where; y = 0.7069x + 2.5016. Correlations with the number of Regional Ecosystems (RE) present in each subregion were assessed as a measure of heterogeneity, and to the percentage of rainforest area in protected areas (%PA), Forestry and those areas combined (%PAandF). Where multiple tests were undertaken the Bonferroni correction was applied.

In order to further investigate relationships and similarities among subregion community floras we then calculated pairwise dissimilarity matrices among the subregions. We used the unweighted Bray—Curtis metric to calculate dissimilarity in species composition using presence absence subregion community data and Regional Ecosystem (RE) composition, as well as dissimilarity in family composition where the number of taxa within the families represented in each subregion was used; these were undertaken using the Primer 6.1.5 software package [70]. Phylogenetic dissimilarity (*Dpw*) matrix and mean nearest phylogenetic neighbour distance (*Dnn*) between communities [66] was calculated in PICANTE as per Swenson [68]. PD dissimilarity (unifrac PD dissimilarity) among subregion pairs was calculated using a Bray-Curtis type metric [12] using the Unifrac package [69].We undertook non-metric multidimensional scaling analysis to investigate relationships and patterns among subregions for each of these dissimilarity measures, using the Primer 6.1.5 program [70] and compared the differences in relationships revealed in the 2D and 3D plots. To quantify the relationships among the different measures of subregion dissimilarity, pairs of dissimilarity matrices were tested for correlation using a Mantel’s test equivalent and the nonparametric Spearman’s Rank correlation coefficient (RELATE function) in Primer 6.1.5 [70]. Because five subregions contained much less area and diversity of rainforest and potentially skewed results, the correlations and pattern analyses were conducted both with and without these subregions.

## Results

### Diversity

**Fig 3:**
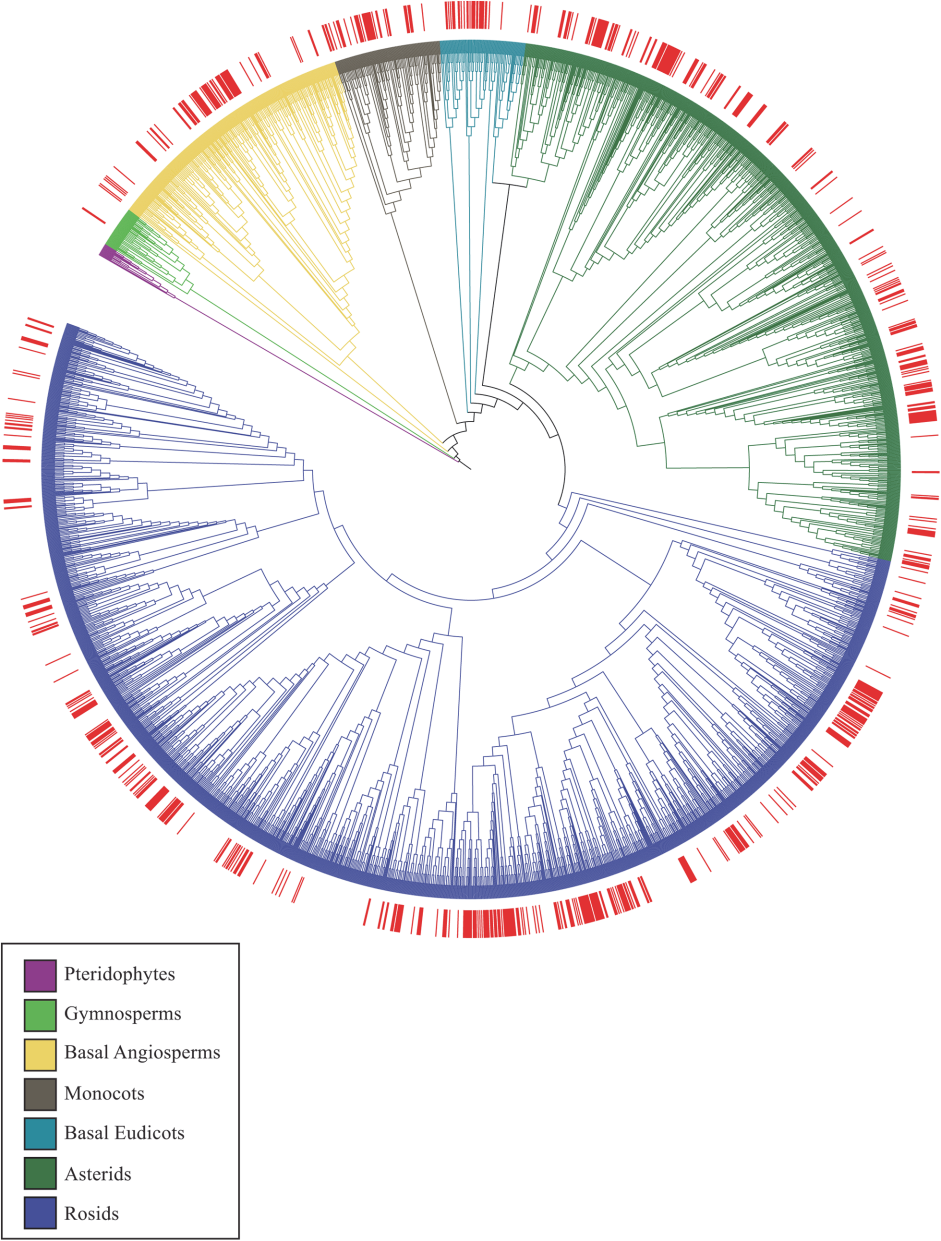
Graphical representation derived from the Rooted dated constrained Phylogenetic tree of the combined tropical rainforest data set used to construct the SE Queensland tree. The phylogeny is presented as an undated cladogram. The SE Queensland rainforest community is indicated by solid bars at the tips of the tree. The tree branches are coloured to indicate major plant groupings.

**Fig 4:**
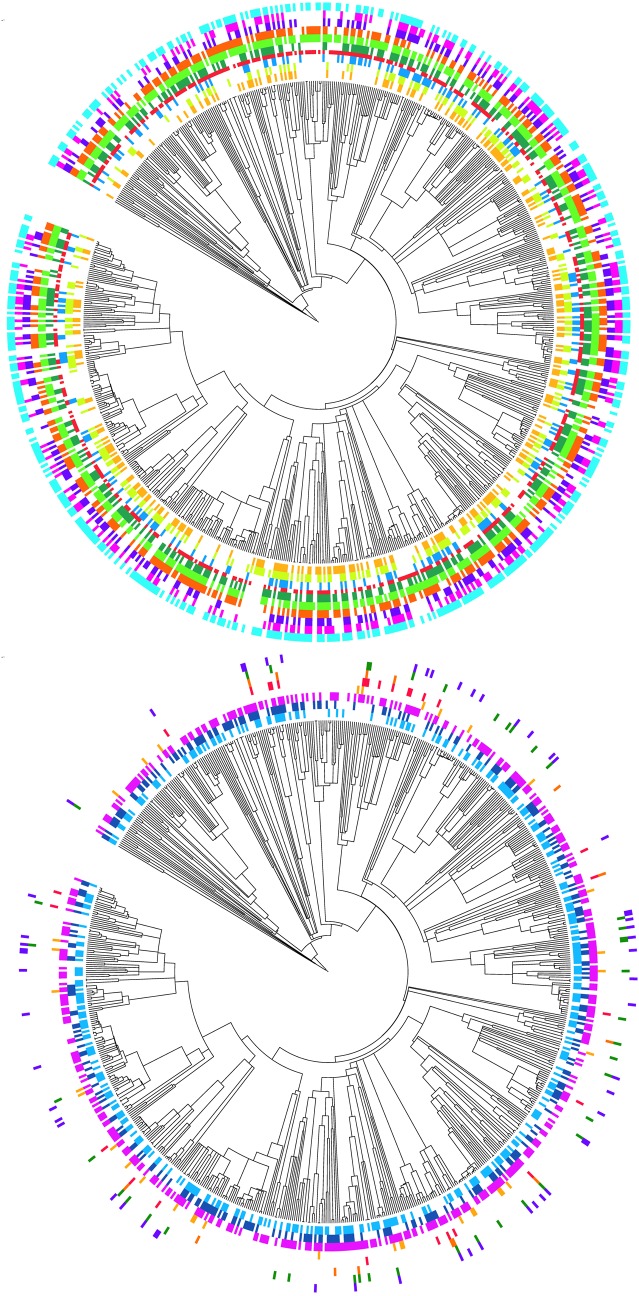
Graphical representation derived from the Rooted dated constrained Phylogenetic tree for SE Queensland based on 3 marker DNA barcode data showing the composition of each subregion by different coloured bars corresponding to the species presence in the subregion. The phylogeny is drawn as a undated cladogram. Subregions are labelled from inner to outer Top tree: 11.14, 11.18, 11.31, 12.1, 12.2, 12.3, 12.4, 12.5, 12.6, 12.7. Bottom tree: 12.8, 12.9, 12.10, 12.11, 13.1, 13.2, 13.3, 11.22.

Our study sampled 86% of the known SE Queensland rainforest flora of 870 species excluding epiphytic orchids and ferns with all 111 families represented (Table 1). Fig. 3 depicts the phylogenetic position of the SE Queensland species within the phylogeny of species drawn from the Smithsonian Institution collection of rainforest permanent plots from several countries. SE Queensland phylogenetic diversity spans most major branches of the tree and samples broadly from the pool of rainforest species diversity, although some groups are not represented in the region. The mean nearest taxon distance (MNTD) for the whole SE Queensland community is quite low, but within each subregion it is higher, indicating that sister species generally are not occupying the same subregions (Table 1, Fig. 4).

**Table 1:** Summary of South East Queensland rainforest diversity according to subregions.

**Sub region**	**Subregion Location**	**SR**	**Fam**	**PD**	**MPD**	**NRI**	**MNTD**	**NTI**
11.14	Marlborough Plains	283	76	17430.25	326.99	^C^*-3.16	79.61	0.11
11.18	Mount Morgan Ranges	295	74	17480.47	318.79	^C^ *-5.92	79.79	0.51
11.22	Banana—Auburn Ranges	56	29	5244.15	324.51	-1.43	137.90	-0.83
11.31	Eastern Darling Downs	214	67	14361.88	332.51	-1.30	86.07	-0.47
12.1	Scenic Rim	475	101	24857.60	344.44	^E^*3.29	63.37	0.32
12.2	Moreton Basin	400	89	21823.49	335.12	-1.07	69.92	0.61
12.3	Burringbar—Conondale Ranges	547	105	26367.14	341.30	^E^*2.10	59.34	0.32
12.4	Sunshine Coast—Gold Coast Lowlands	445	97	23294.85	342.03	1.88	64.54	-0.06
12.5	Brisbane—Barambah Volcanics	310	80	19108.56	331.42	^C^ *-2.05	84.69	*2.21
12.6	South Burnett	284	78	18207.81	331.64	-1.91	83.71	1.06
12.7	Gympie Block	484	92	24099.47	332.88	^C^ *-2.61	61.56	-0.25
12.8	Burnett—Curtis Coastal Lowlands	365	87	20783.40	330.74	^C^ *-2.66	77.50	1.93
12.9	Great Sandy	314	80	18516.00	344.47	^E^*2.08	73.34	-0.67
12.10	Burnett—Curtis Hills and Ranges	442	96	23637.80	334.77	-1.47	70.21	*1.95
12.11	Woodenbong	35	26	3655.04	320.78	-1.47	141.25	-1.72
13.1	Stanthorpe Plateau	28	20	3329.25	336.82	-0.07	174.64	-0.80
13.2	Tenterfield Plateau	14	11	2136.97	338.24	0.00	258.24	0.70
13.3	Nandewar Northern Complex	42	26	4575.86	324.68	-1.20	155.69	-0.54
**Total**	**SEQueensland rainforest**	**752**	**111**	**31711.69**	**337.89**		**49.967**	

Where SR is the rainforest species richness; Fam the number of families present; PD the phylogenetic diversity; MPD the mean PD of taxa within the subregion; MNTD mean nearest taxon distance within the subregion; NRI net relatedness index within the subregion; NTI nearest taxon index within the subregion indicate the probability that a subregion deviates significantly from random (2 tailed test) where significant values are indicated *.

^E^ indicates significant taxonomic evenness (species more distant than random).

^C^ indicates significant taxonomic clustering (species more similar than random ^C^).

**Fig 5:**
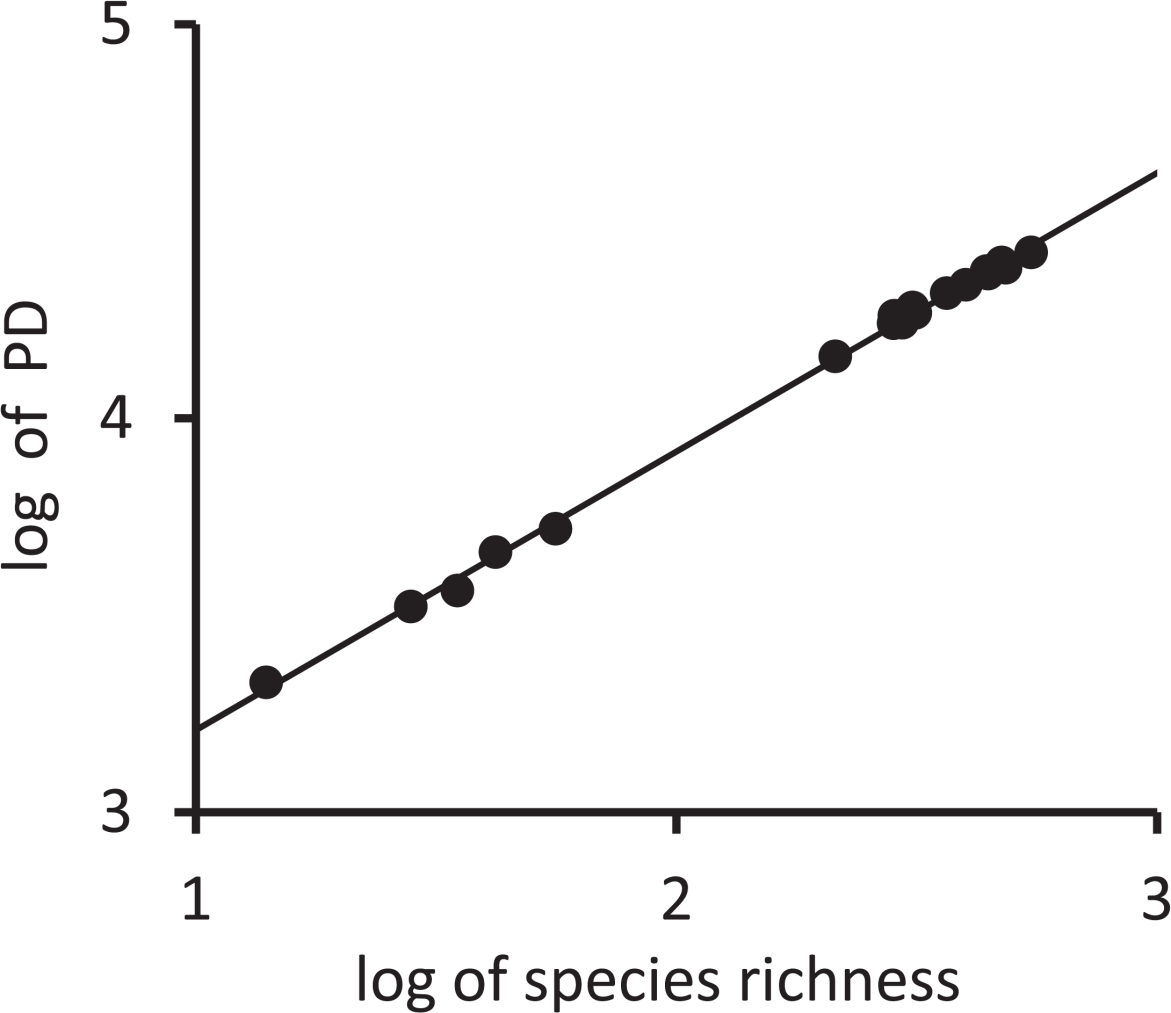
Plot of log transform of species richness (SR) against the log transform of PD for each SE Queensland subregion where R² = 0.9985.

**Fig 6:**
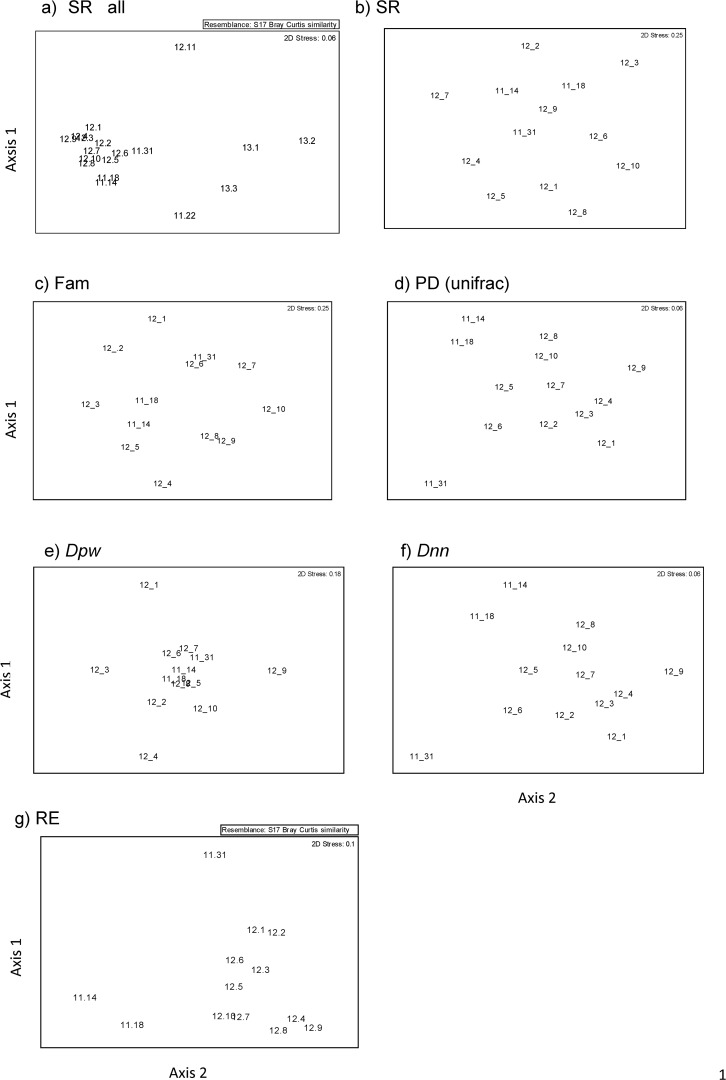
Comparison of non-metric Multidimensional Scaling analyses undertaken comparing relationships among SE Queensland subregions rainforest flora where low diversity outliers excluded for all except a) unweighted species composition with all subpopulations; b) unweighted species composition; c) family composition used Bray-Curtis dissimilarity matrices (2D outputs); d) using PD UniFrac dissimilarity matrix; e) *Dnn* dissimilarity; f) *Dpw* dissimilarity; g) RE composition. The ordination output on the first two axes is shown in the plots.

The subregions containing the highest diversity (12.3,12.7,12.1) were predominantly upland subregions; 12.3 Burringbar-Connondale Ranges with highest species and family richness (SR, Fam) as well as the highest phylogenetic diversity (PD), followed by 12.7 the Gympie Block and 12.1 Scenic Rim (Table 1). The log of PD was strongly linearly correlated with the log of species richness (SR: R² = 0.9985; Fig. 5). Whilst SR, Fam, PD, and MNTD were significantly (p< 0.05) correlated as expected, they did reveal some important distinctions. For example, whilst 12.1 has lower SR it has a higher PD and more families represented compared to 12.7 (Table 1). Further, the species in 12.3 and 12.1 were significantly less closely related than expected by chance (phylogenetic evenness / over dispersion) with significant positive NRI values, whereas species in 12.7 contained significantly more closely related taxa than expected by chance (phylogenetically clustered) with significant negative NRI values (Table 1). The fourth most species rich (SR) subregion 12.4 Sunshine Coast—Gold Coast Lowlands had more families represented than 12.7 but had a lower PD value with a non- significant NRI value (Table 1). The outlying south western Subregions 12.11, 11.22, 13.1, 13.2, 13.3 had the lowest diversity (SR, FAM, PD, Table 1, Figs. 1 and 2). However, these regions contained species from widely differing taxonomic groups and hence have much greater MNTD than the more species rich subregions (Table 1). They were visibly distinctly separate from all other subregions in all MDS clusters (e.g. Fig. 6) except the (*Dpw*) MDS cluster and these subregions contained much smaller areas of remnant and preclearing rainforest compared to all other regions (Tables 1 and 2) therefore they were excluded and cluster and correlation analyses redone to elucidate finer scale patterns.

**Table 2:** Summary of South East Queensland subregion rainforest extent preclearing and remnant vegetation (as of 2009).

**Subregion**	**Area(Ha)**	**Pre RF(Ha)**	**Rem RF (Ha)**	**%Rem RF**	**Pre RF% Subregion**	**RemRF% sub**	**RE’s**	**% PA**	**%PAandF**
11_14	1216837	16995	11351	66.79	1.40	0.93	9	33	39
11_18	1275970	58901	18167	30.84	4.62	1.42	9	9	15
11_22	1547556	110163	16736	15.19	7.12	3.32	12	1	8
11_31	1698060	25896	3057	11.80	1.53	0.18	10	1	9
12_1	228692	66127	41728	63.10	28.92	18.25	16	76	76
12_2	784969	46797	4807	10.27	5.96	0.61	12	8	8
12_3	535410	83876	38370	45.75	15.67	7.17	14	52	63
12_4	365498	11972	4015	33.54	3.28	1.10	11	17	21
12_5	806778	72836	41990	57.65	9.03	5.20	18	51	75
12_6	563866	126512	23474	18.55	22.44	4.16	17	46	68
12_7	859024	119615	49601	41.47	13.92	5.77	15	44	70
12_8	706910	29660	4692	15.82	4.20	0.66	8	13	20
12_9	362412	9200	7346	79.85	2.54	2.03	7	85	91
12_10	1031742	42315	34726	82.07	4.10	3.37	16	65	74
12_11	2846	845	59	6.98	29.69	2.07	3	0	0
13_1	137941	16	16	100.00	0.01	0.01	1	100	100
13_2	7456	na	na	na	na	na	0	na	na
13_3	629398	234	108	46.15	0.04	0.02	1	0	0
**Total**	**12761365**	**821960**	**300243**	**336.5**	**6.44**	**2.35**	**41**	**47**	**60**

Total area of each subregion (Area), Pre clearing area of rainforest (Pre RF),Area of remnant rainforest (Rem RF), Percentage of preclearing rainforest remaining (% Rem RF) and the preclearing percentage of the subregion area occupied by rainforest (Pre RF% Subregion), the percentage of the subregion area occupied by remnant rainforest (RemFR %sub) and the number of Regional Ecosystem types (RE’s) found, the percentage of remnant rainforest in protected areas (%PA) and % remnant rainforest in combined PA and forestry areas (%PAandF) are given.

In contrast to expectations, diversity (SR, PD, Fam) was not significantly (p>0.05) correlated with the area of rainforest within subregions either preclearing or remnant remaining (nor Ln of these areas). However diversity was significantly (p<0.05) positively correlated with the percentage of the subregion occupied by remnant rainforest (SR Rho = 0.577, PD Rho = 0.637; Fam Rho = 0.553). PD was also significantly correlated (Rho = 0.582, p = 0.037) with the preclearing rainforest area as a percentage of the total area of the subregion. Diversity (PD, SR) was also significantly correlated with the number of Regional Ecosystems (RE) present when all subregions were analysed but this relationship was not significant when the depauperate subregions were excluded. This suggests that vegetation heterogeneity is linked to diversity but it does not adequately explain variation among more species rich areas.

### Patterns

Several subregions 11.14, 11.18, 12.5, 12.7 and12.8 contained species that were significantly more closely related than expected (NRI) based on randomisation of the complete SE Queensland rainforest taxa dataset, suggesting some phylogenetic clustering as might be expected for more recently colonised or disturbed rainforest areas. These subregions form two geographically adjacent groups; 12.5, 12.7 and 12.8 are geographically clustered and associated with subcoastal ranges and are just north of the cluster of subregions with significant phylogenetic evenness (Fig. 2). Species in 12.5 were on average significantly phylogenetically more distant than expected given their species richness indicating taxonomic distinctiveness (NTI; Table 1).

The NRI values for 11.14 and 11.18 indicate significant phylogenetic clustering (Table 1), consistent with disturbed or more recently colonised areas. The subregions 11.14, 11.18 were identified as distinctive outliers in the ordination based on uniFrac PD dissimilarity. *Dnn* as well as their RE composition supported this. The rest were broadly clumped except for 11.31 which is perhaps the most distinctly outlying of all subregions (Fig. 6). The distance matrices of these three metrics were significantly correlated for these subregions (Rho = 0.7, p = 0.01). The Subregions 11.14 and 11.18 are the most northern subregions geographically so it is not surprising that they cluster and show some taxonomic differentiation from the more southern subregions (Figs. 1 and 2). The phylogenetic trees clearly show that there are some taxonomic groups with long branches present in these subregions that are absent from the more southern diverse subregions, notably *Terminalia* and *Macropteranthes* genera from the tropical family Combretaceae and *Memecylon* from the pan tropical family Memecylaceae (Fig. 4). However, the family analysis shows that these northern subregions are not distinctive at the family level (Fig. 6C). There is a distinct north south latitudinal contrast revealed by PD dissimilarity with greatest pairwise values observed between the most southern subregion 12.1 and the northern subregions 11.18 and 11.14 (PD dissimilarity Unifrac = 0.599; 0.542). There is also an east west effect where the next most distant subregions based on PD analyses were the most easterly 12.9 and most inland westerly subregion 11.31 (PD dissimilarity unifrac = 0.535; Fig. 6D).

The subregions with the smallest PD dissimilarity (Unifrac) were 12.3 and adjacent 12.4 (0.155) with 12.3 and 12.1 the next most similar based on PD dissimilarity (0.164). However, the analysis using MPD distance (*Dpw*) identified subregions 12.1, 12.3, 12.4 and 12.9 as outliers while the other subregions were clumped (Fig. 6F). These subregions 12.1, 12.3, 12.4, 12.9, contained significantly more distantly related taxa than expected by chance (NRI) hence displaying phylogenetic evenness and 12.1, 12.3, 12.4 contained the highest diversity (SR, Fam, PD, Table 1). These subregions are contiguous with each other and represent the areas covering the southeastern upland (12.1), subcoastal ranges (12.3) and adjacent coastal and subcoastal lowlands and foothills 12.4, 12.9 (Figs. 1 and 2) with the coastal lowland areas containing lower diversity than the upland areas. The upland areas of 12.7 are predicted to have been climatically more stable maintaining moist refugial conditions [36] and together with their high diversity are phylogenetically distinctive in containing several geographically restricted genera in the Proteaceae which has a Gondwanan distribution [71] (Fig. 4). Subregions 12.1, 12.4 and 12.10 are the most distinctive outliers at the family level (Fig. 6). They represent a north south gradient in higher diversity rainforest blocks (Figs. 1 and 2) with higher family richness (Table 1); however, they are significantly (p<0.05) different from one another in terms of the families represented (Fig. 5). So while Family dissimilarity was significantly correlated (p<0.05) with Unifrac PD dissimilarity and *Dnn* they highlighted different relationships. Taxa in subregion 12.10 were on average significantly (p<0.05) more phylogenetically distant than expected due to chance given their species richness indicating taxonomic distinctiveness (NTI; Table 1).

The subregion vegetation type (RE) composition was significantly (p = 0.001; Rho = 0.7) correlated with Unifrac PD dissimilarity and *Dnn* dissimilarity suggesting environmental heterogeneity as indicated by habitat diversity has led to selection of more distant taxa. Subregions with more similar RE composition whilst not more similar in terms of species composition contain groups of taxa that are more related/similar suggesting selection for certain traits. Subregion 11.31 stands out again as significantly (p<0.05) different in terms of the RE communities present and as being phylogenetically distinct from other subregions (Fig. 6). The coastal subregions 12.4, 12.8 and 12.9 are similar in their RE composition (Figs. 2 and 6).

### Conservation

Whilst overall 47 percent of the area of remnant rainforest remaining in SE Queensland is currently in Protected Areas, within each subregion the percentage of its rainforest that is within protected areas varies enormously, with some subregions conserving little or none of the rainforest within it (Table 2). The percentage of rainforest within a subregion Protected Areas (%PA) was not significantly correlated with any measures of diversity found within the subregion’s rainforest estate (SR, Fam, PD, MNTD, MPD). The percent of rainforest within protected areas was significantly correlated with the percentage of the subregion occupied by rainforest RemRF% sub (Rho = 0.775), weakly with the area of remnant rainforest and most strongly with the percent of preclearing rainforest area remaining (Rho = 0.883), but it was not correlated with the area of rainforest preclearing. However, the percent of forestry area was significantly (p<0.05) correlated with the preclearing rainforest area (Rho = 0.575).

We will consider conservation priorities from two important perspectives. The first prioritises places that are quite diverse (e.g. have high total PD). Such locations not only may have many different lineages, but also may be places that are relatively intact or healthy, so providing important functions and ecosystem services. The second criterion takes a regional perspective, prioritising those places that are highly complementary, or distinctive, relative to other places in the region.

When considering the conservation needs for the most diverse subregions, we find that there is considerable variation in level of protection. In the most diverse subregion 12.3, there is currently 52% of remnant rainforest area in protected areas which is much less that the 76% of 12.1, hence the more diverse rainforest in 12.3 is much less well protected (Table 2). The third most diverse subregion, 12.7 is less well protected with 44% of the remnant rainforest protected in reserves and a significant portion 26%, within state forest or timber reserve designated in part for forestry activities (Table 2). The next most diverse subregions, 12.4 and 12.10, contain very similar SR and numbers of families with 12.4 slightly higher than 12.10 for these values (Table 1). Whilst 12.4 contains some of the highest diversity in the region and significant phylogenetic evenness/dispersion only 17% of the remnant rainforest is conserved in protected areas (Table 2). However, 12.10 has a higher PD value and clustered as distinct at the family level and it has significantly greater NTI values (nearest taxon index) than expected compared to random. Thus PD and barcoding analysis have provided additional evidence that the 12.10 subregion is of great conservation significance for the SE Queensland rainforest species pool. Fortunately subregion 12.10 is much better conserved with 65% of remnant rainforest present in protected areas and 9% in state forest (Table 2).

When the distinctiveness of the sub-regions in the ordination space is examined, different conclusions are derived. Priority for 12.3 relative to 12.1 is not well-justified; 12.3 shares PD with 12.4. In contrast, 12.1 is quite distinctive (Fig. 6D). The case also can be made that 12.7 is a concern, but by the same logic, even more dramatic is the need for more protected area within 12.2. In contrast, subregions 11.14 and 11.18 were highly distinctive in their phylogenetic composition. It is noteworthy therefore that the rainforest in these areas is poorly conserved (Table 2). The ordination (Fig. 6D) has high explanatory power as a representation of PD similarities and differences among the sub-regions (as indicated by the low “stress” value). The ordination also is well-justified as a summary of environmental space, given that major north-south and east-west (coastal vs upland) gradients are apparent. It therefore provides a good basis for conservation priority setting and assessments.

## Discussion

### Diversity

The subtropical rainforest of South East Queensland contain less than half of the number of plant species compared with the wet tropical rainforest of Northern Queensland [49]. However, as predicted the phylogenetic diversity of this rainforest estate is much higher than might be expected based on area and fragmentation history. Pleistocene glacial and interglacial fluctuations, extinctions and lags in postglacial recolonization of climatically disrupted areas can contribute to lower diversities [72], [41]. The results are consistent with our current understanding of Australian biogeographic history where the ancient mesic flora contracted with subsequent species extinctions particularly over past Pleistocene climatic cycles [36], [46]. SE Queensland has a large number of families represented (111) compared to the total number of species with deep phylogenetic roots present. By comparison the recent phylogenetic study in Panama [18] included a similar number of species (792) but these represented only 68 families. Our results found that even in this more southern rainforest area there was a wide representation of global tropical rainforest taxa and families which is consistent with the predictions of strong patterns of dispersal of mesic tropical flora into Australia from the north dating from 20 MYA [46]. These results confirm that while Australian rainforests may not contain as many species as mega diverse tropical regions elsewhere, they contain comparable levels of phylogenetic diversity. Where a phylogeny for an area contains relatively few species relative to families, species richness is thought to be an inaccurate surrogate for phylogenetic diversity [73], [9]. Whilst this study found species richness was correlated with phylogenetic diversity (PD) the subregions were not ranked in the same order of diversity. In SE Queensland rainforest sister species are generally not found in the same subregions and this is consistent with ancient lineages that have contracted to refugia where they may have undergone allopatric speciation [37], [46], [36].

The subregions containing the highest diversity were predominantly near coastal upland areas. Crisp et al. [47] identified the importance of elevation on species richness and endemism for refugial function. Cowling and Lombard [74] also found diversity to be correlated with elevation in more mesic areas in South Africa. In contrast Kooyman et al. [35] found elevation accounted for little of the variance in community phylogenetic structure or trait variation across local and regional scales in Queensland rainforests. The areas with the lowest diversity were located in the drier south western subregions where the area occupied by rainforest is much less. Comparable environmental effects on diversity were found in South Africa [74]. Spasojevic and Suding [75] found evidence that Phylogenetic diversity increased with increasing resource availability, while Zobel et al. [76] found that species diversity mirrors the abundance of habitats in evolutionary history. Historically mesic forest retracted eastwards in Australia and present day rainforest distribution reduces along a rainfall gradient [46], [35], [36]. The low diversity subregions were phylogenetically distinct from other more diverse rainforest areas by all measures and contain phyogenetically distant species assemblages (MDNT) suggesting a depauperate sample of rainforest diversity.

Laurance et al. [27] reported that species richness of many different taxa decreased in smaller patches in the Amazonian rainforest. In Australia, rainforest ecosystems account for a high proportion of species diversity despite their small area of occupancy [46], [36]. However, in contrast to expectations, subregion rainforest area, either remnant or preclearing, was not correlated with measures of diversity (SR, PD) in SE Queensland. The relationship between species richness and area is thought to be in part explained by larger areas sampling more species due to higher numbers, covering greater habitat diversity and containing more biogeographical provinces [77], [78], [28]. Our study compared biogeographical provinces (subregions) for their diversity so reduced the effect of one of these factors. Other studies of SE Queensland rainforest also found no significant relationship between diversity and habitat area [79]. Turner and Tjørve [78] suggest that habitat diversity influences species diversity at all spatial scales. In our study diversity (PD, SR) was significantly correlated with the number of Regional Ecosystems (RE) present suggesting that habitat diversity may contribute more to diversity in SE Queensland rainforests than the area of rainforest. This is consistent with results of Weber et al. [36]; however, it does not explain the variation in diversity among the more species rich areas well.

Subregional rainforest phylogenetic diversity was significantly positively correlated with the percentage of the subregion occupied by rainforest both preclearing and remnant and was more sensitive to this than species richness. Given that diversity was not correlated with rainforest area *per se* this correlation suggests present rainforest connectivity may be an important contributor to diversity. Fragmentation and metapopulation theories predict that greater connectivity will maintain higher diversity within habitat patches [22], [80], [81]. The results suggest that perhaps the fragmentation impacts on PD are older than recent anthropogenic clearing. Other authors have suggested that evolutionary history and ecosystem productivity are the most important correlates of species richness patterns [82], [83], [28]. Weber et al. [36] found that processes affecting diversity, including current rainfall, rainforest area, and topographic complexity, explained 58% of variation in plant-weighted endemism and taxa.

### Patterns

Zhang et al. [18] and Fine and Kembel [44] found evidence of non-random phylogenetic structure in tropical rainforest ecosystems but varied in their explanation of the causes of the patterns of diversity. Byrne et al. [46] predicted phylogenetic structuring at fine, as well as broad geographical scales in Australian rainforests from long-term persistence through multiple climatic cycles. Our results found both non-random and random patterns in community phylogenetic relationships and considerable differences among subregions within SE Queensland in their phylogenetic composition and distinctiveness. Kooyman et al. [40] found significant phylogenetic evenness for rainforest in the Australian Wet Tropics (N Queensland) but not in the subtropical regions studied which were to the south of our study in northern New South Wales.

In contrast to Kooyman et al. [35], [40] who used a phylogenetic supertree, our subregional study found evidence of potential subtropical rainforest refugia displaying phylogenetic evenness/dispersion. Four adjacent subregions (12.1, 12.3, 12.4, 12.9) covering the southeastern upland and subcoastal ranges and adjacent coastal and subcoastal lowlands and foothills displayed significant phylogenetic evenness but were also phylogenetically the most similar subregions. The adjacent coastal lowland areas contained lower diversity than the upland areas. These subregions are consistent with the centres of endemism proposed by Weber et al. [36] for subtropical rainforest in Australia. The upland areas are predicted to have been climatically more stable maintaining moist refugial conditions [36]. The presence of basal lineages, such as the rainforest Proteaceae family, in these subregions support this [71], [46], [40]. While the presence of evenness /dispersion is predicted for refugial areas, those which have expanded from these are predicted to show phylogenetic clustering [40]. The Great Sandy Region (12.9) would be predicted to have phylogenetic clustering being a geologically younger white sand substrate, but this area which is identified by Weber et al. [36] as being part of a refugial area, had significant evenness. Our results are in contrast to those of Fine and Kembel [44] who found taxa to be phylogenetically clustered in white-sand forests in Peru consistent with environmental filtering.

Yessoufou et al. [84] highlighted that phylogenetic clustering of plant communities has been attributed to several mechanisms including, habitat filtering [85] disturbance [86], facilitation [87], competition and biotic interchange [88]. The Gympie Block (12.7), one of the subregions with the highest phylogenetic diversity, is phylogenetically clustered indicating the species are more similar to each other than expected. There were two groups of adjacent subregions that were phylogenetically clustered; the southern group (12.5, 12.7and 12.8) are just north of the subregions with significant phylogenetic evenness. Fine and Kembel [95] suggested that their observed phylogenetic clustering in the Amazon was consistent with habitat specialisation. Whereas, Kooyman et al. [35], [40] suggested phylogenetic clustering on the more southern end of subtropical rainforest was in recolonised areas. Whilst the coastal subregions may have expanded from refugial areas in the more upland Gympie Block the high diversity in this subregion is less consistent with post glacial rainforest expansion.

The second group of adjacent subregions that are phylogenetically clustered (11.14 and 11.18) are the most northern subregions. In this study they group as phylogenetically different from the more southern subregions as well as having a distinctive set of vegetation types (RE’s) present. Thus these results are more consistent with effects of invasion of more tropical taxonomic groups notably from the tropical families Combretaceae and Memecyclaceae across the current ‘dry’ barrier north of Rockhampton often referred to as the St Lawrence gap [89], [90]. However, they also would be consistent with an effect of habitat specialisation [44] as they share similar distinctive vegetation communities. This is largely due to climatic differences following increasing temperature and decreasing rainfall gradients. Drier sites support shorter rainforest stands with increasingly deciduous canopies, fewer epiphytes and many thorny or spiny species suggesting strong selection pressures [45], [42]. Zhang et al. [18] showed that both spatial and environmental distances are significant correlates of phylogenetic beta diversity. In this study, Subregions with more similar vegetation community (RE) composition are more similar in their phylogenetic composition suggesting selection for phylogenetically conserved traits. The results are consistent with broad predictions of higher diversity and ancient lineages conserved in subtropical refugia in the moist Southeastern uplands but provide finer detail. However, the influences leading to other phylogenetic patterns, especially clustering, seem to be more variable and complex. More detailed analyses of the SE Queensland rainforest estate are needed to clarify the nature of these patterns. The use of DNA barcode data to generate PD measures of richness and dissimilarity has enabled better understanding of the nature and distribution of biodiversity and has shown to be more sensitive than species richness.

### Conservation

It is widely recognised that we should aim to ensure that all ecosystem and habitat types are represented within regional conservation strategies [2], [4], [24]. Soutullo et al. [91] in their global assessment of the convention on biodiversity (CBD) in 2008 identified that Australia had not met its target obligation. Our study shows that with 47 percent of the remaining area of subtropical rainforest currently within the Protected Areas network this significant high biodiversity ecosystem is relatively well protected and meets its 2020 obligations for this significant area of biodiversity [1]. This region is an important test for Australia to show international leadership in conservation of rainforest in the more difficult areas of contrasting high land use demands that are typical of its developing tropical neighbours [92] [93]. It contrasts with the Australian wet tropics rainforest which has had considerable conservation planning efforts [94] and is under less pressure from land use conflicts and urban expansion than South East Queensland which is one of the areas of greatest population growth in Australia [5].

Whilst some question their effectiveness, many still argue that protected areas are the most effective mechanism for global biodiversity conservation [95], [91].The results of this study show that the setting aside of protected areas has been an effective measure to conserve rainforest as it was strongly correlated with the percentage of preclearing rainforest area remaining in a subregion. However, the size of the protected area estate was not correlated with the preclearing rainforest area, this in contrast with the area of rainforest set aside for forestry which was correlated with the preclearing rainforest area. This reflects global patterns that place economic benefits above biodiversity benefits [92], [93]. The conversion of significant areas of former forestry land tenure to the Protected Area Network has clearly has a positive impact of SE Queensland rainforest conservation.

This study found that most species are contained within the most diverse 4–5 subregions combined (12.3, 12.1, 12.7, 12.4, 12.10) these with the exception of 12.4 (17%) are relatively well protected (44–76%) in the National Reserve System (NRS). However, the results also show that the remaining diversity of species is geographically and environmentally dispersed. The northern subregions (11.14, 11.18) are both phylogenetically and ecologically distinct, PD analysis has clarified the importance of these areas. Subregion 11.8 has only 9% in the NRS and only a third of the preclearing area remains; this subregion thus deserves high priority for expansion of the Protected Area Network.

The distinctiveness of these sub-regions was revealed well in the ordination of Fig. 6D. Faith et al. [12] reviewed the many similar studies that have used PD dissimilarities (many of these are microbial and use UniFrac). Our results match these in providing high explanatory power and revealing good correspondence with environmental gradients. We note that, of the ordination analyses explored here, only those using dissimilarity measures based on Bray- Curtis dissimilarities (including UniFrac) are well justified as a basis for conservation planning [12]. Faith et al. [12] noted that most studies have not carried out any further analyses using the ordination space, and they suggested that a method developed for analysis of species-level ordinations could provide powerful insights for genomics-based environmental or ordination spaces. This method, “environmental diversity” (ED), can provide conservation assessments and priority setting based on the environmental space. ED has been used in this way for conventional species-level ordinations (see Faith et al. [96]) but has not yet been applied for phylogenetic diversity.

This same space can be used for systematic conservation planning that balances biodiversity conservation with economic and other needs of society. The basic ED method (for examples see Faith et al [96]) provides estimates of complementarity values- expected gains or losses in diversity- when a “site” (a point in the ordination space) is gained or lost. These gains can be balanced with “costs” reflecting competing needs of society. This simple analysis would be informative for our environmental space if we imagined total gains or losses of sub-regions. For example, if we assumed that rainforest in all sub-regions except 12.7 and 12.3 were protected, ED would identify 12.7 as the higher priority for protection (reflecting the gap it fills in the environmental space).

The potential for applications of the ED approach is well highlighted by our findings that the ordination (Fig. 6D) has good correspondence with one or more environmental gradients and has low stress value. The low stress value implies that ED priority setting based on gaps, such as those described above, will indeed reflect gains/losses in phylogenetic diversity. Further, the link to important environmental gradients suggests that these gains/losses or complementarity values may be indicative of values for other taxonomic groups responding to variation in these same gradients (see Faith et al. [96]). Thus, through such analyses, DNA barcoding may provide a biodiversity surrogate framework for the region. A caveat is that our simple description of ED above which imagined total gains/losses of subregions. In reality, we will be changing the percent area protected for one or more subregions. It is well-established that percent-area targets for subregions, such as those discussed here, can be used most effectively for regional planning if we take the relationships among the subregions into account [97]. The ordination (Fig. 6D) provides this required information about relationships; subregions closer together in the space share more phylogenetic diversity, Extended ED methods [96] use this information to allow us to assess changes in percent area protected for one or more bioregions.

Further, our finding in this study of a very good fit for a power curve relationship between species number and PD (Fig. 5) supports this novel approach. Within a subregion, we can expect a species-area relationship so that fractional area translates to a predicted fractional number of species. The relationship between species number and PD means that we can in turn convert this to a PD area curve within the bioregions, such that fractional change in area within a given bioregion translates into a predicted fractional PD. Future work will explore this approach. As noted in the Introduction, a limitation in such planning is the availability of phylogenies. Integration of DNA barcoding into this framework, along the lines outlined here can provide a dramatic gain in our capacity for effective conservation planning.

Hubbell [98] found most diversity in the Amazonian rainforest was due to rare species which are not randomly distributed. Phylogenetic diversity studies in several tropical rainforest systems have also found non-random patterns [44], [18] confirming that conservation in rainforests needs to be aimed at maximising phylogenetic diversity rather than simply maximising the area conserved. Our study (SR, PD) along with the study by Weber et al. [36] found that Australian subtropical rainforest plant diversity is not strongly correlated with area. This is in keeping with current knowledge that the distribution of diversity at the landscape level is determined by many interacting factors, area being only one of these [19], [20]. However, many conservation planning programs make the SAR assumption and use it as the basis of calculations for cost effectiveness [99]. This study clearly shows that targeting areas based on spatial diversity patterns, ecological gradients and historical biogeography may be more effective. Refugial areas are predicted to have been climatically more stable over geological time and hence might be predicted to be more stable under future climate change [100], [36]. The subregions with higher diversity were generally associated with centres of endemism identified as potential rainforest refugia [36] and would have higher conservation priority.

Currently the Protected Area estate is more effective at conserving subtropical rainforest in the wettest areas but our results have demonstrated evidence that rainforest diversity will be better conserved by conserving a greater diversity of vegetation types (RE) which will capture a greater phylogenetic diversity of species. Vegetation mapping has often been used as a surrogate for conservation planning as it can be mapped on large scales [4]. Thus these results in part support the RE approach to conservation planning in Queensland as a surrogate for environmental heterogeneity.

Conservation of biodiversity is more politically successful when it is seen as providing benefits to humans [100], [101], [102]. Several authors have suggested that conserving PD may be an efficient way to capture a diversity of attributes which may prove useful [101], [102], [103]. Janzen [104] argued that DNA barcoding should be adopted into the inventory to assess and document tropical rainforest biodiversity. This study demonstrates an application for biodiversity assessment and conservation planning that has benefited from this approach. Costion et al. [11] showed how barcodes can determine species numbers accurately and so can be used to supplement rainforest inventory data and methods. The additional data gained on phylogenetic relatedness adds value to this approach especially where taxonomy is not well defined or field identification tools limited. This study identified the areas of highest priority for biodiversity conservation and utilised PD as well SR to ensure greater capture of biodiversity incorporating higher order phylogenetic sampling of biodiversity.

## Supporting Information

S1 Dataset Supplemental data for rainforest phylogeny: outlines the exact dates used and the references from which the age is from—it also identifies the exact NODE on the phylogeny through the most recent common ancestor of two taxa that span the clade being dated. (DOCX)

S1 Output Example output from RAxML analysis. (PDF)

S1 Table Species names, Genbank Accession numbers and Queensland Herbarium (BRI) collector numbers. (DOCX)
