# Supplementary material for: Mapping Biodiversity and Setting Conservation Priorities for SE Queensland’s Rainforests Using DNA Barcoding
Source: PLoS One. 2015 Mar 24;10(3):e0122164. doi: 10.1371/journal.pone.0122164 (PMC4372436; doi:10.1371/journal.pone.0122164)
Supplement: S1 Dataset — (DOCX) [file pone.0122164.s001.docx]

**S3 Dataset:** Supplemental data for rainforest phylogeny : outlines the exact dates used and the references from which the age is from - it also identifies the exact NODE on the phylogeny through the most recent common ancestor of two taxa that span the clade being dated.

Actual dates used for calibration with PATHd8:

Angiosperms: 250myo used as Fixed age in program (from reference above)

node for angiosperms defined as most recent common ancestor between *Illicium* and *Gnetum*.

Magnoliales: 112myo minimum Age constraint

Reference: B. Mohr and M. Bernardes de Oliveira. 2004. *Endressinia brasiliana*, a

Magnolialean Angiosperm from the Lower Cretaceous Crato Formation (Brazil).

International Journal of Plant Sciences 165(6):1121-1133.

node for Magnoliales defined as MRCA: *Virola sebifera* and *Magnolia splendens*

Sapindales: 55.8myo minimum Age constraint

Reference: S. R. Manchester (2001)

Leaves and fruits of *Aesculus* (Sapindales) from the Paleocene of North America

International Journal of Plant Sciences, 162: 985-998.

node defined as MRCA: *Serjania mexicana* and *Acer mono*

Ericales: 89.3myo, minimum Age constraint

Reference: K. C. Nixon and W. L. Crepet. 1993.

Late Cretaceous fossil flowers of Ericalean affinity.

American Journal of Botany 80(6):616-623

node defined as MRCA: *Kalmia latifolia* and *Ardisia standleyana*

*Fabales: 90.3myo minimum age constraint

reference: Koenen, E. et al. 2013. Exploring the temnpo of species diversification in

legumes. South African J. Bot. 89: 19-30.

node defined as MRCA: *Pterocarpus rohrii* and *Inga nobilis*

*Myrtales: 65 m.y. old

Reference:Crepet, W. L. et al. 2004, Friis, E. M., & Gandolfo, M. A. 2004.

Fossil evidence and phylogeny: The age of major angiosperm clades based on mesofossil and

macrofossil evidence from Cretaceous deposits. American J. Bot. 91: 1666-1682.

node defined as MRCA: *Eugenia reinwardtiana* and *Vochysia ferruginea*

Malvales 33.9 minimum age constraint

Reference: Manchester, SR (1999)

Biogeographical relationships of North American Tertiary floras

Annals of the Missouri Botanical Garden, 472-522.

node defined as MRCA: *Hibiscus tiliaceus* and *Grewia latifolia*

Malpighiales 89.3 minimum age constraint

Reference: Crepet and Nixon, 1998

node defined as MRCA: *Bridelia tomentosa* and *Byrsonima crassifolia*

Fagales: 93.5 minimum age constraint

References: Batten, 1981; Kedves, 1989; Pacltová, 1966

node defined as MCRA: *Quercus rubra* and *Betula costata*

Dipsacales 33.9 minimum age constraint.

Reference: Manchester and Donoghue, 1995

node defined as MRCA: *Viburnum prunifolium* and *Lonicera japonica*

Apiales 37.2 minimum age constraint

Reference: Manchester, 1999

node defined as MRCA: *Schefflera heptaphylla* and *Pittosporum glabratum*
