# Supplementary material for: Mapping Biodiversity and Setting Conservation Priorities for SE Queensland’s Rainforests Using DNA Barcoding
Source: PLoS One. 2015 Mar 24;10(3):e0122164. doi: 10.1371/journal.pone.0122164 (PMC4372436; doi:10.1371/journal.pone.0122164)
Supplement: S1 Output — (PDF) [file pone.0122164.s002.pdf]

```
CIPRES_THREADSPP=6
CIPRES_NP=5
running:
 mpirun -hostfile /opt/torque/aux//1646874.trestles-fe1.sdsc.edu -np 5  raxmlHPC-HYBRID -T 6 -s infile -n result -g constraint.tre -q part -p 12345 -k -m GTRCAT -f d -N 6

This is RAxML MPI Process Number: 3

This is RAxML MPI Process Number: 2

This is RAxML MPI Process Number: 4

This is RAxML MPI Process Number: 1

This is RAxML MPI Process Number: 0



IMPORTANT WARNING: Sequences Abies_magnifica and Abies_procera are exactly identical


IMPORTANT WARNING: Sequences Antidesma_japonicum_var_acutisepalum and Antidesma_japonicum_var_densiflorum are exactly identical


IMPORTANT WARNING: Sequences Araucaria_cunninghamii_129308486 and Araucaria_cunninghamii_var_cunninghamii are exactly identical


IMPORTANT WARNING: Sequences Betula_costata and Betula_ermanii are exactly identical


IMPORTANT WARNING: Sequences Betula_costata and Betula_platyphylla are exactly identical


IMPORTANT WARNING: Sequences Distyliopsis_dunnii and Distylium_myricoides are exactly identical


IMPORTANT WARNING: Sequences Drypetes_longifolia and Drypetes_pendula are exactly identical


IMPORTANT WARNING: Sequences Populus_koreana and Populus_ussuriensis are exactly identical


IMPORTANT WARNING: Sequences Ulmus_davidiana and Ulmus_laciniata are exactly identical

IMPORTANT WARNING
Found 9 sequences that are exactly identical to other sequences in the alignment.
Normally they should be excluded from the analysis.


IMPORTANT WARNING
Found 435 columns that contain only undetermined values which will be treated as missing data.
Normally these columns should be excluded from the analysis.


Just in case you might need it, a mixed model file with
model assignments for undetermined columns removed is printed to file part.reduced
Just in case you might need it, an alignment file with
sequence duplicates and undetermined columns removed is printed to file infile.reduced

This is the RAxML Master Pthread

This is RAxML Worker Pthread Number: 1

This is RAxML Worker Pthread Number: 2

This is RAxML Worker Pthread Number: 3

This is RAxML Worker Pthread Number: 4

This is RAxML Worker Pthread Number: 5

This is the RAxML Master Pthread

This is RAxML Worker Pthread Number: 5

This is RAxML Worker Pthread Number: 4

This is RAxML Worker Pthread Number: 1

This is RAxML Worker Pthread Number: 3

This is RAxML Worker Pthread Number: 2

This is the RAxML Master Pthread

This is RAxML Worker Pthread Number: 1

This is RAxML Worker Pthread Number: 2

This is RAxML Worker Pthread Number: 3

This is RAxML Worker Pthread Number: 5

This is RAxML Worker Pthread Number: 4

This is the RAxML Master Pthread

This is RAxML Worker Pthread Number: 1

This is RAxML Worker Pthread Number: 5

This is RAxML Worker Pthread Number: 4

This is RAxML Worker Pthread Number: 3

This is RAxML Worker Pthread Number: 2

This is the RAxML Master Pthread
```

```
This is RAxML Worker Pthread Number: 1

This is RAxML Worker Pthread Number: 2

This is RAxML Worker Pthread Number: 3

This is RAxML Worker Pthread Number: 4

This is RAxML Worker Pthread Number: 5


This is RAxML version 7.6.6 released by Alexandros Stamatakis on July 9 2013.

With greatly appreciated code contributions by:
Andre Aberer (HITS)
Simon Berger (HITS)
Nick Pattengale (Sandia)
Wayne Pfeiffer (SDSC)
Akifumi S. Tanabe (NRIFS)


Alignment has 19939 distinct alignment patterns

Proportion of gaps and completely undetermined characters in this alignment: 96.91%

RAxML rapid hill-climbing mode

Using 3 distinct models/data partitions with joint branch length optimization


Executing 6 inferences on the original alignment using 6 user-specifed trees

All free model parameters will be estimated by RAxML
ML estimate of 25 per site rate categories

Likelihood of final tree will be evaluated and optimized under GAMMA

GAMMA Model parameters will be estimated up to an accuracy of 0.1000000000 Log Likelihood units

Partition: 0
Alignment Patterns: 526
Name: rbcl
DataType: DNA
Substitution Matrix: GTR



Partition: 1
Alignment Patterns: 1179
Name: matK
DataType: DNA
Substitution Matrix: GTR



Partition: 2
Alignment Patterns: 18234
Name: psbA
DataType: DNA
Substitution Matrix: GTR




RAxML was called as follows:

raxmlHPC-HYBRID -T 6 -s infile -n result -g constraint.tre -q part -p 12345 -k -m GTRCAT -f d -N 6


Partition: 0 with name: rbcl
Base frequencies: 0.270 0.217 0.225 0.288

Partition: 1 with name: matK
Base frequencies: 0.295 0.182 0.162 0.362

Partition: 2 with name: psbA
Base frequencies: 0.254 0.244 0.246 0.257

Inference[0]: Time 13792.891568 CAT-based likelihood -384237.142867, best rearrangement setting 15
Inference[8]: Time 15750.912933 CAT-based likelihood -384227.280671, best rearrangement setting 25
Inference[2]: Time 15870.003067 CAT-based likelihood -384207.867298, best rearrangement setting 25
Inference[4]: Time 15927.043687 CAT-based likelihood -384209.106597, best rearrangement setting 25
Inference[6]: Time 16026.572295 CAT-based likelihood -384226.041640, best rearrangement setting 25
Inference[7]: Time 12899.997699 CAT-based likelihood -384207.214595, best rearrangement setting 20
Inference[1]: Time 15698.245708 CAT-based likelihood -384213.468550, best rearrangement setting 20
Inference[5]: Time 14199.388043 CAT-based likelihood -384167.356007, best rearrangement setting 15
Inference[9]: Time 15222.737898 CAT-based likelihood -384158.576957, best rearrangement setting 20
Inference[3]: Time 27700.946074 CAT-based likelihood -384236.483206, best rearrangement setting 25


Conducting final model optimizations on all 10 trees under GAMMA-based models ....

Inference[2] final GAMMA-based Likelihood: -400810.310584 tree written to file /projects/ps-ngbt/backend/trestles_workspace/NGBW-JOB-RAXMLHPC2_TGB-
F6494F199FFA42E2ACB7F7694EE4B3D1/RAxML_result.result.RUN.2
Inference[8] final GAMMA-based Likelihood: -400828.807595 tree written to file /projects/ps-ngbt/backend/trestles_workspace/NGBW-JOB-RAXMLHPC2_TGB-
F6494F199FFA42E2ACB7F7694EE4B3D1/RAxML_result.result.RUN.8
Inference[9] final GAMMA-based Likelihood: -400697.052181 tree written to file /projects/ps-ngbt/backend/trestles_workspace/NGBW-JOB-RAXMLHPC2_TGB-
F6494F199FFA42E2ACB7F7694EE4B3D1/RAxML_result.result.RUN.9
Inference[3] final GAMMA-based Likelihood: -400734.783396 tree written to file /projects/ps-ngbt/backend/trestles_workspace/NGBW-JOB-RAXMLHPC2_TGB-
F6494F199FFA42E2ACB7F7694EE4B3D1/RAxML_result.result.RUN.3
Inference[4] final GAMMA-based Likelihood: -400810.995633 tree written to file /projects/ps-ngbt/backend/trestles_workspace/NGBW-JOB-RAXMLHPC2_TGB-
F6494F199FFA42E2ACB7F7694EE4B3D1/RAxML_result.result.RUN.4
Inference[5] final GAMMA-based Likelihood: -400730.088458 tree written to file /projects/ps-ngbt/backend/trestles_workspace/NGBW-JOB-RAXMLHPC2_TGB-
F6494F199FFA42E2ACB7F7694EE4B3D1/RAxML_result.result.RUN.5
Inference[0] final GAMMA-based Likelihood: -400734.844043 tree written to file /projects/ps-ngbt/backend/trestles_workspace/NGBW-JOB-RAXMLHPC2_TGB-
F6494F199FFA42E2ACB7F7694EE4B3D1/RAxML_result.result.RUN.0
```

```
Inference[1] final GAMMA-based Likelihood: -400793.083945 tree written to file /projects/ps-ngbt/backend/trestles_workspace/NGBW-JOB-RAXMLHPC2_TGB-
F6494F199FFA42E2ACB7F7694EE4B3D1/RAxML_result.result.RUN.1
Inference[6] final GAMMA-based Likelihood: -400825.296672 tree written to file /projects/ps-ngbt/backend/trestles_workspace/NGBW-JOB-RAXMLHPC2_TGB-
F6494F199FFA42E2ACB7F7694EE4B3D1/RAxML_result.result.RUN.6
Inference[7] final GAMMA-based Likelihood: -400746.609795 tree written to file /projects/ps-ngbt/backend/trestles_workspace/NGBW-JOB-RAXMLHPC2_TGB-
F6494F199FFA42E2ACB7F7694EE4B3D1/RAxML_result.result.RUN.7


Starting final GAMMA-based thorough Optimization on tree 9 likelihood -400697.052181 ....

Final GAMMA-based Score of best tree -400689.082236

Program execution info written to /projects/ps-ngbt/backend/trestles_workspace/NGBW-JOB-RAXMLHPC2_TGB-F6494F199FFA42E2ACB7F7694EE4B3D1/RAxML_info.result
Best-scoring ML tree written to: /projects/ps-ngbt/backend/trestles_workspace/NGBW-JOB-RAXMLHPC2_TGB-F6494F199FFA42E2ACB7F7694EE4B3D1/RAxML_bestTree.result

Overall execution time: 63876.454239 secs or 17.743460 hours or 0.739311 days
```
