# Supplementary material for: Mapping Biodiversity and Setting Conservation Priorities for SE Queensland’s Rainforests Using DNA Barcoding
Source: PLoS One. 2015 Mar 24;10(3):e0122164. doi: 10.1371/journal.pone.0122164 (PMC4372436; doi:10.1371/journal.pone.0122164)
Supplement: S1 Table — (DOCX) [file pone.0122164.s003.docx]

| Species | *matK* Accession | *trnH-psbA* Accession | *rbcLa* Accession | Collector Number |
| --- | --- | --- | --- | --- |
| *Abrophyllum ornans* | KM894583 | KM895126 |  | P.Dufourq MFNP |
| *Abutilon auritum* | KM894532 | KM895088 | KM895630 | P.I.Forster PIF39016 |
| *Abutilon micropetalum* | KM894746 |  | KM895898 | P.I.Forster PIF39006 |
| *Abutilon oxycarpum* | KM894618 |  | KM895732 | G. Smyrell GS-52-5 |
| *Acacia bakeri* | KM894854 | KM895317 | KM896024 | Guymer D2 |
| *Acacia disparrima* | KM894481 | KM895056 |  | shapcottAcDi1-1 |
| *Acacia fasciculifera* | KM894903 | KM895355 | KM896087 | G. Smyrell GS 41-7 |
| *Acacia harpophylla* | KM894642 |  | KM895764 | G. Smyrell GS-49-9 |
| *Acacia maidenii* | KM894773 | KM895266 | KM895932 | G. Smyrell GS-31-6 |
| *Acacia melanoxylon* | KM894938 |  | KM896131 | P.Dufourq MFNP |
| *Acacia oshanesii* | KM894869 | KM895329 | KM896041 | ShapcottAcOs1-1 |
| *Acalypha capillipes* | KM894987 |  | KM896197 | Young |
| *Acalypha eremorum* |  |  | KM895890 | ShapcottAcEr1-1 |
| *Acalypha nemorum* |  |  | KM896172 | Guymer D30 |
| *Acalypha sp. Big Scrub* |  |  | KM895839 | P.I.Forster PIF38784 |
| *Acmena ingens* | KM894537 | KM895091 | KM895634 | P.I.Forster PIF38554 |
| *Acmena smithii* |  | KM895396 | KM896155 | P.Dufourq MMSF |
| *Acradenia euodiiformis* | KM894715 | KM895219 | KM895855 | P.I.Forster PIF38704 |
| *Acronychia baeuerlenii* | KM894963 |  | KM896168 | W.J.McDonald 2012-02-003 |
| *Acronychia imperforata* | KM894907 | KM895358 | KM896092 | P.I .Forster PIF38994 |
| *Acronychia laevis* | KM894770 | KM895263 | KM895928 | ShapcottMBB AcLa1-1 |
| *Acronychia oblongifolia* | KM894614 | KM895146 | KM895726 | P.I.Forster PIF38566 |
| *Acronychia octandra* | KM894968 |  |  | P.I.Forster PIF38703 |
| *Acronychia pauciflora* | KM894972 |  | KM896176 | P.I.Forster PIF38290 |
| *Acronychia pubescens* | KM894824 | KM895239 | KM895991 | P.I.Forster PIF38698 |
| *Acronychia suberosa* | KM894946 |  | KM896143 | W.J.McDonald 2012-02-005 |
| *Acronychia wilcoxiana* | KM894623 | KM895155 | KM895737 | G.Smyrell GS-48-5 |
| *Acrotriche aggregata* |  | KM895222 | KM895862 | Smyrell;Kupsch |
| *Actephila bella* |  |  | KM895942 | G. Smyrell GS41-6 |
| *Actephila grandifolia* | KM894677 |  | KM895812 | P.I.Forster PIF38733 |
| *Actephila lindleyi* |  |  | KM896158 | ShapcottAcLi1-1 |
| *Agathis robusta* |  | KM895417 | KM896189 | P.Dufourq BBBP |
| *Aglaia brownii* | KM894487 |  | KM895577 | G.Smyrell GS-6-1 |
| *Aglaia elaeagnoidea* | KM894515 |  | KM895610 | G.Smyrell GS-11-1 |
| *Aidia racemosa* | KM894976 | KM895410 | KM896179 | G.Smyrell GS-8-2 |
| *Ailanthus triphysa* | KM894426 | KM895009 | KM895503 | P.I.Forster PIF38533 |
| *Akania bidwillii* | KM894919 |  | KM896105 | G.Smyrell GS-7-1 |
| *Alangium villosum* | KM894723 | KM895226 | KM895866 | ShapcottAlVi1-1 |
| *Alchornea ilicifolia* |  | KM895331 | KM896046 | P.Dufourq KFNPlb |
| *Alchornea thozetiana* | KM894712 |  | KM895852 | G.Smyrell GS-24-1 |
| *Alectryon connatus* | KM894470 | KM895347 | KM895560 | ShapcottAlCo1-1 |
| *Alectryon coriaceus* |  |  | KM895519 | ShapcottAlCo1-1 |
| *Alectryon diversifolius* | KM894801 | KM895279 | KM895965 | G.Smyrell GS-49-4 |
| *Alectryon oleifolius* | KM894493 | KM895063 | KM895583 | BJ3 |
| *Alectryon ramiflorus* |  |  | KM895834 | G.Smyrell GS-17-1 |
| *Alectryon reticulatus* | KM894865 | KM895161 | KM896035 | Halford QM584 |
| *Alectryon semicinereus* | KM894679 |  |  | G. Smyrell GS-44-3 |
| *Alectryon subdentatus* |  | KM895294 | KM895984 | G.Smyrell GS-4-1 |
| *Alectryon tomentosus* | KM894598 | KM895133 | KM895707 | P.Dufourq NCCP |
| *Alloxylon pinnatum* | KM894771 | KM895264 | KM895929 | P.I.Forster PIF38708 |
| *Alocasia brisbanensis* | KM894991 |  | KM896204 | ShapcottAlBr1-1 |
| *Alphitonia excelsa* |  | KM895386 | KM896142 | P.Dufourq KFNP23 |
| *Alphitonia petriei* | KM894554 | KM895104 |  | ShapcottAlPe1-1 |
| *Alpinia arundelliana* |  |  | KM895823 | R.Etherington BSF |
| *Alpinia caerulea* |  |  | KM896031 | P.Dufourq MFNP |
| *Alstonia constricta* | KM894783 | KM895270 |  | G.Smyrell GS-22-1 |
| *Alyxia magnifolia* | KM894996 |  | KM896209 | G.Smyrell GS42-1 |
| *Alyxia ruscifolia* | KM894942 | KM895383 | KM896137 | P.Dufourq BBBP |
| *Alyxia sharpei* |  |  | KM895911 | G.Smyrell GS-10-2 |
| *Alyxia stellata* | KM894975 |  |  | G.Smyrell GS-27-1 |
| *Amorphospermum antilogum* | KM894918 |  | KM896104 |  |
| *Amyema cambagei* |  |  | KM896045 |  |
| *Amyema quandang* |  |  | KM895821 | BJ15 |
| *Amylotheca dictyophleba* |  | KM895092 | KM895635 | ShapcottAmDi1-1 |
| *Aneilema acuminatum* |  |  | KM895556 | ShapcottAnAc1-1 |
| *Anopterus macleayanus* | KM894751 |  |  | P.I.Forster PIF38707 |
| *Anthocarpa nitidula* | KM894644 |  | KM895768 | P.Dufourq GPP22 |
| *Aphananthe philippinensis* | KM894881 | KM895338 | KM896058 | P.Dufourq BC04 |
| *Aphanopetalum resinosum* |  |  | KM895541 | B. Jeffers |
| *Apophyllum anomalum* | KM894542 | KM895096 | KM895646 | G. Smyrell GS-49-5 |
| *Araucaria bidwillii* |  |  | KM895985 | P.Dufourq MFNP |
| *Araucaria cunninghamii* |  |  | KM895744 | P.Dufourq BBBP |
| *Archidendron grandiflorum* | KM894986 | KM895420 | KM896196 | G.Smyrell GS-7-2 |
| *Archidendron muellerianum* | KM894601 | KM895244 | KM895710 | W.J.McDonald 2012-01-013 |
| *Archidendropsis thozetiana* |  | KM895048 | KM895558 | ShapcottArTh1-1 |
| *Archirhodomyrtus beckleri* | KM894647 | KM895175 | KM895772 | P.DufourqBFP |
| *Archontophoenix alexandrae* | |  | KM895600 | G. Smyrell GS-IN-1 |
| *Archontophoenix cunninghamiana* | KM894651 |  | KM895776 | Shapcott GFMR AcCu1-1 |
| *Ardisia bakeri* |  |  | KM896048 | P.I.Forster PIF38734 |
| *Argophyllum nullumense* | KM894822 | KM895298 | KM895989 | P.I.Forster PIF38711 |
| *Argyrodendron actinophyllum* | | KM895045 | KM895552 | ShapcottArAc1-1 |
| *Argyrodendron sp. Kin Kin* | KM894719 |  | KM895859 | P.Dufourq BFP |
| *Argyrodendron trifoliolatum* |  | KM895315 | KM896019 | P.Dufourq GPP |
| *Arytera dictyoneura* | KM894466 | KM895044 | KM895551 | G.Smyrell GS-14-1 |
| *Arytera distylis* | KM894441 | KM895022 | KM895521 | P.I.Forster PIF38360 |
| *Arytera divaricata* | KM894909 | KM895362 | KM896095 | P.Dufourq GPP |
| *Arytera foveolata* | KM894533 |  | KM895631 | G.Smyrell GS-3-1 |
| *Arytera microphylla* |  |  | KM895808 | Halford QM580 |
| *Atalaya hemiglauca* | KM894492 |  | KM895582 | BJ4 |
| *Atalaya multiflora* | KM894959 |  | KM896164 | Guymer D8 |
| *Atalaya rigida* | KM894779 | KM895268 |  | G. Smyrell GS-54-2 |
| *Atalaya salicifolia* | KM894629 |  | KM895746 | G.Smyrell GS-3-2 |
| *Atractocarpus benthamianus* | KM894580 |  | KM895691 | P.I.Forster PIF38697 |
| *Atractocarpus chartaceus* | KM894440 |  | KM895520 | P.I.Forster PIF38524 |
| *Auranticarpa rhombifolia* | KM894886 | KM895344 | KM896062 | P.I.Forster PIF38553 |
| *Austrobuxus swainii* |  | KM895380 | KM896126 | P.I.Forster PIF38723 |
| *Austrosteenisia blackii* | KM894641 | KM895172 | KM895762 | G.Smyrell GS-1-3 |
| *Austrosteenisia glabristyla* | KM894625 |  | KM895740 | P.Dufourq GPP04 |
| *Backhousia angustifolia* | KM894680 | KM895200 | KM895815 | G. Smyrell GS-49-8 |
| *Backhousia citriodora* | KM894439 | KM895020 | KM895518 | ShapcottBaCi1-1 |
| *Backhousia kingii* |  | KM895350 | KM896076 | P.I.Forster PIF15063 ex cult. |
| *Backhousia leptopetala* | KM894758 | KM895256 | KM895913 | P.I.Forster PIF38999 |
| *Backhousia myrtifolia* | KM894781 |  | KM895944 | P.Dufourq KFNPlb |
| *Backhousia oligantha* | KM894720 | KM895223 | KM895863 | Young |
| *Backhousia sciadophora* | KM894434 | KM895015 | KM895513 | G. Smyrell GS-IN-15 |
| *Backhousia subargentea* | KM894511 |  | KM895603 | ShapcottChSu1-1 |
| *Baloghia inophylla* |  |  | KM896145 | P.Dufourq KFNPlb15 |
| *Baloghia marmorata* | KM894665 |  | KM895795 | P.I.Forster PIF38364 |
| *Barklya syringifolia* |  | KM895357 | KM896091 | ShapcottBaSy1-1 |
| *Beilschmiedia elliptica* | KM894995 | KM895424 | KM896207 | P.Dufourq KFNPlb16 |
| *Beilschmiedia obtusifolia* | KM894462 | KM895040 | KM895545 | P.Dufourq GPP03 |
| *Berberidopsis beckleri* | KM894573 |  | KM895683 | P.I.Forster PIF38694 |
| *Beyeria viscosa* |  |  | KM896141 | P.I.Forster PIF38584 |
| *Boehmeria macrophylla* | KM894844 | KM895312 | KM896014 | P.I.Forster PIF38717 |
| *Bosistoa medicinalis* | KM894628 | KM895159 | KM895745 | G. Smyrell GS-IN-22 |
| *Bosistoa pentacocca* | KM894859 | KM895322 | KM896029 | G. Smyrell GS-IN-23 |
| *Bosistoa transversa* | KM894827 |  | KM895993 | ShapcottBoTr1-1 |
| *Bouchardatia neurococca* | KM894969 |  | KM896173 | ShapcottBoNe1-1 |
| *Brachychiton acerifolius* | KM894654 | KM895179 | KM895780 | B. Jeffers |
| *Brachychiton australis* | KM894768 | KM895262 | KM895925 | P.I.Forster PIF7502 |
| *Brachychiton bidwillii* | KM894683 | KM895201 | KM895818 | G.Smyrell GS-9-1 |
| *Brachychiton discolor* | KM894970 | KM895402 |  | P.Dufourq BMNP |
| *Brachychiton populneus* | KM894664 | KM895189 |  | P.Dufourq BMNP07 |
| *Brachychiton rupestris* | KM894632 | KM895162 | KM895749 | ShapcottBrRu1-1 |
| *Brachychiton sp. Ormeau* |  |  | KM896042 | ShapcottBrOm1-1 |
| *Breynia oblongifolia* | KM894857 | KM895319 | KM896027 | ShapcottBrOb1-1 |
| *Bridelia exaltata* |  | KM895390 | KM896147 | G.Smyrell GS-31-4 |
| *Bridelia leichhardtii* |  | KM895102 | KM895660 | ShapcottBiLe1-1 |
| *Bursaria incana* |  | KM895425 | KM896208 | G. Smyrell GS-49-10 |
| *Cadellia pentastylis* | KM894648 |  |  | P.I.Forster PIF38673 |
| *Caesalpinia bonduc* | KM894702 |  | KM895844 | G.Smyrell GS-24-2 |
| *Caesalpinia nitens* |  | KM895025 | KM895525 | Young |
| *Caesalpinia scortechinii* | KM894454 | KM895032 | KM895534 | ShapcottCaSc1-1 |
| *Caesalpinia subtropica* |  |  | KM896188 | ShapcottCasu1-1 |
| *Calamus muelleri* | KM894935 |  | KM896129 | ShapcottCaMu1-1 |
| *Calanthe triplicata* | KM894885 |  | KM896061 | P.Dufourq MFNP |
| *Caldcluvia paniculosa* |  |  | KM896191 | P.Dufourq MMSF |
| *Callerya australis* | KM894496 |  | KM895586 | P.I.Forster PIF38831 |
| *Callerya megasperma* |  |  | KM895548 | G .Leiper s.n. |
| *Callicarpa pedunculata* | KM894575 |  | KM895685 | G.Smyrell GS-40-8 |
| *Callicoma serratifolia* | KM894952 |  | KM896153 | Smyrell;Kupsch |
| *Callitris glaucophylla* |  |  | KM895763 | P.I.Forster PIF38598 |
| *Callitris macleayana* |  |  | KM895869 | P.I.Forster PIF38844 |
| *Canarium australasicum* |  |  | KM895524 | W.J.McDonald 2012-01-005 |
| *Capparis arborea* | KM894943 | KM895286 | KM896139 | P.Dufourq GPP |
| *Capparis lasiantha* | KM894551 |  | KM895658 | BJ15 |
| *Capparis loranthifolia* | KM894709 |  | KM895849 | G. Smyrell GS-49-6 |
| *Capparis mitchellii* | KM894690 | KM895205 | KM895827 | BJ10 |
| *Capparis ornans* | KM894945 |  |  | G. Smyrell GS-IN-3 |
| *Capparis sarmentosa* | KM894921 |  | KM896107 | Halford QM583 |
| *Capparis velutina* |  |  | KM895742 | G. Smyrell GS-44-1 |
| *Carallia brachiata* |  |  | KM895564 | G. Smyrell GS-IN-12 |
| *Carissa ovata* | KM894543 | KM895097 | KM895648 | P.I.Forster PIF38587 |
| *Carronia multisepalea* | KM894529 | KM895086 | KM895627 | P.I.Forster PIF38751 |
| *Casearia multinervosa* | KM894433 | KM895169 | KM895512 | G.Smyrell GS-2-1 |
| *Cassia brewsteri* | KM894820 | KM895296 | KM895987 | Guymer D14 |
| *Cassia marksiana* | KM894728 | KM895231 | KM895872 | P.I.Forster PIF38786 |
| *Cassia tomentella* | KM894456 | KM895035 | KM895538 | P.I.Forster PIF38960 |
| *Cassinia subtropica* | KM894666 | KM895190 | KM895796 | P.I.Forster PIF38868 |
| *Cassytha pubescens* |  | KM895073 | KM895595 | G.Smyrell GS-15-7 |
| *Castanospermum australe* | KM894449 | KM895029 | KM895528 | P.Dufourq MMSF |
| *Castanospora alphandii* | KM894422 |  | KM895499 | P.Dufourq GPP |
| *Cayratia acris* | KM894429 | KM895011 | KM895507 | G.Smyrell GS-25-1 |
| *Cayratia clematidea* | KM894979 | KM895414 | KM896184 | P.Dufourq KFNP |
| *Cayratia eurynema* | KM894547 | KM895099 | KM895653 | P.I.Forster PIF38972 |
| *Cayratia saponaria* | KM894766 | KM895260 | KM895921 | ShapcottCaSap1-1 |
| *Celastrus australis* | KM894480 | KM895055 | KM895571 | P.I.Forster PIF38555 |
| *Celastrus subspicata* | KM894924 | KM895372 | KM896110 | P.I.Forster PIF38966 |
| *Celtis paniculata* | KM894667 | KM895191 | KM895798 | P.Dufourq NCCP |
| *Cephalaralia cephalobotrys* | KM894595 | KM895132 | KM895704 | P.Dufourq MMSF |
| *Ceratopetalum apetalum* | KM894747 | KM895248 | KM895900 | B. Jeffers |
| *Chionanthus ramiflorus* | KM894499 |  | KM895589 | G. Smyrell GS-47-1 |
| *Cinnamomum oliveri* | KM894760 |  | KM895915 | G.Smyrell GS-7-3 |
| *Cinnamomum virens* | KM894586 |  | KM895697 | P.I.Forster PIF38696 |
| *Cissus antarctica* | KM894737 | KM895238 | KM895883 | P.Dufourq BC |
| *Cissus hypoglauca* |  | KM895277 | KM895959 | P.I.Forster PIF38389 |
| *Cissus oblonga* | KM894645 |  |  | G.Smyrell GS-8-4 |
| *Cissus sterculiifolia* | KM894694 |  | KM895832 | G.Smyrell GS-7-4 |
| *Citronella moorei* |  |  | KM896008 | W.J.McDonald 2012-02-008 |
| *Citrus australasica* |  |  |  | P.I.Forster PIF38746 |
| *Citrus australis* |  | KM895163 | KM895750 | Guymer D27 |
| *Citrus glauca* | KM894940 |  | KM896135 | BJ6 |
| *Claoxylon australe* |  | KM895276 | KM895957 | ShapcottClAu1-1 |
| *Claoxylon tenerifolium* |  |  | KM896193 | G.Smyrell GS-6-4 |
| *Clausena brevistyla* |  |  | KM895757 | G. Smyrell GS-IN-18 |
| *Clausena smyrelliana* |  | KM895207 | KM895831 | G.Smyrell GS-15-3 |
| *Cleistanthus cunninghamii* |  |  | KM895814 | ShapcottClCu1-1 |
| *Clematicissus opaca* |  | KM895089 | KM895632 | G.Smyrell GS-5-1 |
| *Clematis glycinoides* | KM894604 | KM895137 | KM895713 | G. Smyrell GS-31-8 |
| *Clematis pickeringii* | KM894949 | KM895393 |  | Halford (Moss Garden) |
| *Clerodendrum floribundum* | KM894594 | KM895131 | KM895703 | P.Dufourq NCCP |
| *Coatesia paniculata* | KM894516 | KM895078 | KM895611 | G Smyrell GS-52-1 |
| *Coelospermum paniculatum* | KM894476 |  | KM895568 | P.I.Forster PIF38721 |
| *Coelospermum reticulatum* |  | KM895095 | KM895645 | G. Smyrell GS-31-3 |
| *Commersonia bartramia* | KM894446 | KM895028 |  | P.Dufourq KFNPlb |
| *Commersonia fraseri* | KM894706 |  | KM895847 | ShapcottCoFr1-1 |
| *Cordyline congesta* | KM894513 |  | KM895607 | P.I.Forster PIF38786 |
| *Cordyline manners-suttoniae* | KM894468 |  | KM895557 | G. Smyrell GS-41-5 |
| *Cordyline murchisoniae* | KM894605 |  |  | G. Smyrell GS45-1 |
| *Cordyline petiolaris* | KM894825 |  |  | L. Simmons |
| *Cordyline rubra* | KM894522 |  |  | ShapcottCoRu1-1 |
| *Correa lawrenciana* |  | KM895412 | KM896182 | P.I.Forster PIF38739 |
| *Corynocarpus rupestris* | KM894780 |  | KM895943 | Young |
| *Cossinia australiana* |  | KM895245 | KM895893 | G. Smyrell GS-30-8 |
| *Croton acronychioides* |  |  | KM895770 | P.I.Forster PIF38359 |
| *Croton insularis* | KM894861 |  | KM896033 | G.Smyrell GS-3-3 |
| *Croton lucens* |  |  | KM895756 | G. Smyrell GS-53-5 |
| *Croton mamillatus* |  |  | KM895672 | P.I.Forster PIF38983 |
| *Croton phebalioides* |  |  | KM895663 | P.I.Forster PIF38568 |
| *Croton stigmatosus* |  |  | KM895853 | Guymer D17 |
| *Croton verreauxii* |  | KM895171 | KM895761 | G. Smyrell GS3-4-9 |
| *Cryptocarya bidwillii* | KM894908 | KM895361 | KM896094 | P.I.Forster PIF39011 |
| *Cryptocarya erythroxylon* | KM894802 |  | KM895966 | G.Smyrell GS-7-5 |
| *Cryptocarya floydii* | KM894845 | KM895313 | KM896015 | P.I.Forster PIF38567 |
| *Cryptocarya foetida* | KM894477 | KM895053 | KM895569 | P.I.Forster PIF38387 |
| *Cryptocarya foveolata* | KM894640 | KM895170 | KM895760 | P.I.Forster PIF38747 |
| *Cryptocarya glaucescens* | KM894992 |  | KM896205 | P.Dufourq KFNP |
| *Cryptocarya hypospodia* | KM894582 | KM895125 | KM895693 | G.Smyrell GS-15-4 |
| *Cryptocarya laevigata* | KM894896 | KM895369 | KM896075 | P.Dufourq BFP |
| *Cryptocarya macdonaldii* | KM894765 | KM895258 | KM895919 | P.Dufourq GPP |
| *Cryptocarya meisneriana* | KM894557 | KM895105 |  | P.I.Forster PIF38726 |
| *Cryptocarya microneura* | KM894917 |  | KM896102 | Smyrell;Kupsch |
| *Cryptocarya obovata* |  |  |  | B. Jeffers |
| *Cryptocarya onoprienkoana* | KM894925 | KM895373 | KM896112 | B. Jeffers |
| *Cryptocarya rigida* | KM894697 | KM895304 | KM895836 | W.J.McDonald 2012-01-007 |
| *Cryptocarya sclerophylla* | KM894893 | KM895349 | KM896070 | P.Dufourq MFNP |
| *Cryptocarya sp. World End* |  |  | KM895694 | G. Smyrell GS29-5 |
| *Cryptocarya triplinervis* | KM894467 | KM895047 | KM895555 | P.Dufourq GPP |
| *Cupaniopsis anacardioides* | KM894928 |  | KM896116 | G.Smyrell GS-25-4 |
| *Cupaniopsis baileyana* | KM894962 |  | KM896167 | P.I.Forster PIF38551 |
| *Cupaniopsis flagelliformis* | KM894452 |  | KM895532 | P.I.Forster PIF38771 |
| *Cupaniopsis newmanii* | KM894743 | KM895247 | KM895895 | P.I.Forster PIF38349 |
| *Cupaniopsis parvifolia* | KM894721 | KM895224 | KM895864 | G.Smyrell GS-6-3 |
| *Cupaniopsis serrata* | KM894879 |  | KM896056 | P.Dufourq KFNP |
| *Cupaniopsis shirleyana* |  |  | KM895769 | Guymer D6 |
| *Cupaniopsis simulata* | KM894792 |  | KM895952 | Young |
| *Cupaniopsis sp. Biggenden* |  |  | KM895816 | Young |
| *Cupaniopsis sp. Bulburin* | KM894637 |  |  | G. Smyrell GS-40-7 |
| *Cupaniopsis sp. Wataglin* | KM894767 |  | KM895923 | G.Smyrell GS-9-5 |
| *Cupaniopsis tomentella* | KM894931 |  | KM896121 | PI Forster PIF38958 |
| *Cupaniopsis wadsworthii* | KM894581 | KM895123 |  | G.Smyrell GS-9-5 |
| *Cuttsia viburnea* | KM894489 |  | KM895579 | ShapcottCuVi1-1 |
| *Cyclophyllum coprosmoides* | KM894590 |  | KM895699 | Shapcott CyCo1-1 |
| *Cyclophyllum longipetalum* |  |  | KM896079 | G. Smyrell GS-35-2 |
| *Cynanchum bowmanii* | KM894912 |  | KM896097 | P.I.Forster PIF39012 |
| *Cynanchum carnosum* | KM894862 | KM895324 | KM896034 | Mathieson MTM1228 |
| *Daphnandra tenuipes* |  | KM895109 | KM895674 | W.J.McDonald 2012-01-003 |
| *Davidsonia johnsonii* | KM894752 | KM895252 | KM895905 | W.J.McDonald 2012-01-001 |
| *Decaspermum humile* | KM894754 | KM895254 | KM895908 | P.I.Forster PIF38736 |
| *Decaspermum struckoilicum* | KM894866 | KM895327 | KM896037 | P.I.Forster PIF4984 ex cult. |
| *Deeringia amaranthoides* |  | KM895021 | KM895505 | ShapcottDeAm1-1 |
| *Deeringia arborescens* | KM894915 |  | KM896100 | P.I.Forster PIF38785 |
| *Dendrocnide excelsa* | KM894636 |  | KM895755 | P.Dufourq NCCP |
| *Dendrocnide photinophylla* | KM894978 | KM895413 | KM896183 | ShapcottDePh1-1 |
| *Denhamia bilocularis* |  | KM895237 | KM895882 | P.I.Forster PIF38680 |
| *Denhamia celastroides* | KM894643 |  | KM895766 | P.Dufourq BBBP |
| *Denhamia cunninghamii* | KM894475 | KM895051 | KM895565 | P.I.Forster PIF38669 |
| *Denhamia disperma* | KM894662 |  | KM895793 | Halford |
| *Denhamia parvifolia* | KM894507 |  | KM895597 | G. Smyrell GS-52-3 |
| *Denhamia pittosporoides* | KM894889 | KM895346 | KM896065 | P.I.Forster PIF38681 |
| *Denhamia silvestris* | KM894418 |  | KM895493 | P.I.Forster PIF38833 |
| *Derris involuta* | KM894478 |  | KM895570 | G.Smyrell GS-30-5 |
| *Dianella caerulea* | KM894459 |  |  | P. Dufourq PA |
| *Dinosperma erythrococcum* | KM894899 | KM895351 | KM896081 | G.Smyrell GS-2-2 |
| *Dinosperma melanophloia* |  | KM895259 | KM895920 | G. Smyrell GS-34-3 |
| *Dioscorea transversa* | KM894610 |  | KM895718 | P.Dufourq MFNP |
| *Diospyros australis* |  |  | KM895885 | P.I.Forster PIF38565 |
| *Diospyros fasciculosa* | KM894558 |  | KM895664 | PI Forster PIF38976 |
| *Diospyros geminata* | KM894785 | KM895272 | KM895947 | P.I.Forster PIF38509 |
| *Diospyros humilis* | KM894708 |  | KM895848 | Halford QM582 |
| *Diospyros pentamera* | KM894661 | KM895186 | KM895790 | ShapcottDiPe1-1 |
| *Diospyros sp. Blackall* | KM894546 | KM895098 | KM895652 | G. Smyrell GS-36-5 |
| *Diplocyclos palmatus* | KM894512 |  | KM895605 | ShapcottDiPa1-1 |
| *Diploglottis australis* | KM894672 |  |  | P.Dufourq KFNP |
| *Diploglottis campbellii* | KM894849 |  | KM896020 | ShapcottDiCa1-1 |
| *Dissiliaria baloghioides* | KM894517 |  | KM895613 | P.Dufourq MMSF |
| *Dissiliaria muelleri* |  | KM895100 | KM895656 | ShapcottDiMu1-1 |
| *Doryphora sassafras* | KM894871 |  |  | P.I.Forster PIF38690 |
| *Drymophila moorei* | KM894742 | KM895246 | KM895894 | P.I.Forster PIF38738 |
| *Drypetes deplanchei* |  | KM895046 | KM895553 | G.Smyrell GS-25-3 |
| *Duboisia leichhardtii* | KM894725 | KM895228 | KM895868 | G. Smyrell GS-51-1 |
| *Duboisia myoporoides* | KM894576 | KM895118 | KM895686 | P.I.Forster PIF31832 |
| *Dysoxylum fraserianum* |  |  | KM895939 | ShapcottDyFr1-1 |
| *Dysoxylum gaudichaudianum* | KM894717 |  | KM895857 | G.Smyrell GS-21-1 |
| *Dysoxylum mollissimum* | KM894621 |  | KM895735 | G.Smyrell GS-13-3 |
| *Dysoxylum rufum* | KM894734 |  | KM895879 | ShapcottDyRu1-1 |
| *Echinostephia aculeata* |  | KM895232 | KM895873 | P.I.Forster PIF38779 |
| *Ehretia acuminata* | KM894971 | KM895404 | KM896175 | P.I.Forster PIF38543 |
| *Ehretia membranifolia* | KM894705 | KM895215 | KM895846 | BJ13 |
| *Einadia hastata* | KM894904 |  | KM896088 | B.Jeffers |
| *Einadia nutans* | KM894905 |  | KM896090 | B.Jeffers |
| *Elaeagnus triflora* |  |  | KF496304.1 | ShapcottElTr1-1 |
| *Elaeocarpus eumundi* |  | KM895180 | KM895782 | ShapcottElEu1-1 |
| *Elaeocarpus grandis* |  | KM895392 | KM896149 | P.Dufourq KFNP |
| *Elaeocarpus kirtonii* |  |  | KM895618 | G. Smyrell GS-44-4 |
| *Elaeocarpus obovatus* | KM894521 |  | KM895618 | Halford QM575 |
| *Elaeocarpus reticulatus* | KM894691 |  | KM895828 | P.Dufourq KFNPlb |
| *Elaeodendron australe* | KM894560 |  | KM895666 | P.I.Forster PIF38545 |
| *Elaeodendron melanocarpum* | KM894757 |  | KM895912 | Mathieson MTM1229 |
| *Elattostachys bidwillii* | KM894860 |  | KM896030 | Guymer D37 |
| *Elattostachys nervosa* |  | KM895375 | KM896117 | P.Dufourq KFNP |
| *Elattostachys xylocarpa* |  |  | KM895922 | PI Forster PIF38957 |
| *Embelia australiana* | KM894445 | KM895027 | KM895526 | G.Smyrell GS-6-5 |
| *Emmenosperma alphitonioides* | KM894592 |  | KM895701 | ShawSS4 |
| *Emmenosperma cunninghamii* | KM894630 |  | KM895747 | G.Smyrell GS-13-1 |
| *Endiandra compressa* | KM894837 | KM895309 | KM896009 | Shaw SS3 |
| *Endiandra crassiflora* | KM894797 | KM895076 | KM895958 | W.J.McDonald 2012-02-009 |
| *Endiandra discolor* |  | KM895394 | KM896150 | P.Dufourq GPP |
| *Endiandra floydii* | KM894839 |  |  | P.I.Forster PIF38774 |
| *Endiandra hayesii* | KM894606 | KM895139 | KM895715 | P.I.Forster PIF38841 |
| *Endiandra muelleri* | KM894843 | KM895311 | KM896013 | P.I.Forster PIF38384 |
| *Endiandra pubens* | KM894722 | KM895225 | KM895865 | G Smyrell GS-34-5 |
| *Endiandra sieberi* | KM894944 | KM895385 | KM896140 | G.Smyrell GS-5-2 |
| *Erythrina numerosa* |  | KM895261 | KM895924 | P.I.Forster PIF7623 |
| *Erythrina vespertilio* |  | KM895062 | KM895581 | ShapcottErVe1-1 |
| *Erythroxylum sp. Splityard Creek* | KM894840 |  | KM896011 | P.I.Forster PIF38684 |
| *Eucryphia jinksii* |  | KM895039 | KM895544 | P.I.Forster PIF38770 |
| *Eugenia reinwardtiana* | KM894685 |  | KM895822 | G.Smyrell GS-10-7 |
| *Eupomatia laurina* | KM894868 |  | KM896040 | ShapcottEuLa1-1 |
| *Euroschinus falcatus* | KM894490 | KM895059 | KM895580 | P.I.Forster PIF38511 |
| *Everistia vacciniifolia* | KM894948 |  | KM896146 | G. Smyrell GS-49-13 |
| *Excoecaria dallachyana* | KM894953 |  | KM896154 | G. Smyrell GS-36-1 |
| *Exocarpos latifolius* | KM894916 |  | KM896101 | ShapcottExLa1-1 |
| *Ficus adenosperma* | KM894523 |  | KM895621 | G.Smyrell GS-40-1 |
| *Ficus coronata* | KM894457 | KM895036 | KM895539 | P.Dufourq MFNP |
| *Ficus fraseri* | KM894878 |  | KM896055 | P.Dufourq KFNPlb |
| *Ficus macrophylla* | KM894514 |  | KM895608 | P.Dufourq BMNP |
| *Ficus microcarpa* | KM894588 | KM895128 | KM895698 | ShapcottFiMi1-1 |
| *Ficus obliqua* | KM894609 | KM895141 |  | G.Smyrell GS-14-2 |
| *Ficus opposita* | KM894541 | KM895094 | KM895642 | G.Smyrell GS-3-4 |
| *Ficus racemosa* |  |  | KM896067 | G.Smyrell GS-26-1 |
| *Ficus rubiginosa* | KM894812 | KM895287 | KM895977 | P.I.Forster PIF38296 |
| *Ficus superba* | KM894895 |  | KM896074 | G. Smyrell GS-41-11 |
| *Ficus virens* |  | KM895147 | KM895727 | P.Dufourq KFNPlb |
| *Ficus watkinsiana* |  |  | KM895497 | P.Dufourq BBBP |
| *Fieldia australis* | KM894955 |  |  | P.I.Forster PIF38710 |
| *Fitzalania bidwillii* | KM894420 | KM895006 | KM895495 | G.Smyrell GS-19-1 |
| *Flagellaria indica* |  |  | KM895967 | ShapcottFlIn1-1 |
| *Flindersia australis* |  |  | KM895803 | ShapcottFlAu1-1 |
| *Flindersia bennettiana* | KM894646 | KM895174 | KM895771 | G.Smyrell GS-23-1 |
| *Flindersia collina* |  | KM895220 | KM895860 | P.I.Forster PIF38675 |
| *Flindersia schottiana* | KM894714 |  | KM895854 | ShapcottFlSc1-1 |
| *Flindersia xanthoxyla* |  | KM895181 | KM895783 | P.I.Forster PIF38679 |
| *Floydia praealta* |  | KM895114 | KM895680 | ShapcottFlPr1-1 |
| *Fontainea fugax* |  |  | KM895636 | G. Smyrell GS-IN-16 |
| *Fontainea rostrata* |  |  | KM895804 | G.Smyrell GS-30-7 |
| *Fontainea venosa* | KM894741 | KM895243 | KM895889 | Guymer D3 |
| *Freycinetia scandens* |  |  | KM895960 | P.Dufourq KFNP |
| *Galbulimima baccata* | KM894826 | KM895299 | KM895992 | Shaw SS2 |
| *Geijera parviflora* |  | KM895209 | KM895835 | P.I.Forster PIF38590 |
| *Geijera salicifolia* | KM894569 | KM895111 | KM895677 | G. Smyrell GS-49-1 |
| *Geissois benthamiana* | KM894727 | KM895230 | KM895871 | W.J.McDonald 2012-02-006 |
| *Geitonoplesium cymosum* | KM894608 |  | KM895716 | Halford QM564 |
| *Glochidion ferdinandi* | KM894806 | KM895379 | KM895971 | P.Dufourq MMSF |
| *Glochidion lobocarpum* |  |  |  | P.Dufourq KFNPlb |
| *Glochidion sumatranum* | KM894833 | KM895306 | KM896005 | ShapcottGlSu1-1 |
| *Glossocarya hemiderma* | KM894799 |  | KM895963 | G. Smyrell GS-40-9 |
| *Glycine sp. Marburg* |  |  | Z95552.1 | G. Smyrell GS-48-3 |
| *Gmelina leichhardtii* | KM894778 |  | KM895938 | P.Dufourq BBBP |
| *Gossia acmenoides* | KM894803 |  | KM895968 | ShapcottGoAc1-1 |
| *Gossia bidwillii* | KM894524 |  | KM895622 | ShapcottGoBi1-1 |
| *Gossia fragrantissima* |  | KM895290 | KM895980 | P.I.Forster PIF37845 |
| *Gossia gonoclada* | KM894932 | KM895377 | KM896122 | L. Simmons |
| *Gossia inophloia* |  | KM895411 | KM896181 | Shapcott GoIn1-1 |
| *Graptophyllum excelsum* | KM894495 | KM895065 | KM895585 | ShapcottGrex1-1 |
| *Graptophyllum reticulatum* |  | KM895136 | KM895711 | ShapcottGrRe1-1 |
| *Graptophyllum spinigerum* | KM894455 | KM895034 | KM895536 | G. Smyrell GS-53-3 |
| *Grevillea helmsiae* | KM894591 | KM895129 | KM895700 | G.Smyrell GS-17-2 |
| *Grevillea hilliana* | KM894695 | KM895208 | KM895833 | ShapcottGrHi1-1 |
| *Grevillea robusta* | KM894579 | KM895122 | KM895690 | P.Dufourq CPSF |
| *Grewia latifolia* | KM894682 |  | KM895817 | G.Smyrell GS-30-6 |
| *Guilfoylia monostylis* | KM894538 |  | KM895638 | P.Dufourq NCCP |
| *Guioa acutifolia* | KM894983 |  |  | G. Smyrell GS-55-1 |
| *Guioa semiglauca* | KM894888 | KM895345 | KM896064 | P.Dufourq KFNP |
| *Gymnostachys anceps* | KM894795 | KM895275 |  | ShapcottGyAn1-1 |
| *Gynochthodes canthoides* |  |  | KM895637 | ShapcottMoCa1-1 |
| *Gynochthodes jasminoides* | KM894613 | KM895145 | KM895725 | P.I.Forster PIF38468 |
| *Gyrocarpus americanus* |  |  | KM895937 | P.I.Forster PIF6157 |
| *Halfordia kendack* |  |  | KM895997 | P.I.Forster PIF38467 |
| *Harnieria hygrophiloides* | KM894593 | KM895130 | KM895702 | P.I.Forster PIF38640 |
| *Harpullia alata* | KM894565 | KM895108 | KM895671 | P.I.Forster PIF38702 |
| *Harpullia hillii* | KM894635 | KM895167 | KM895754 | G.Smyrell GS-25-2 |
| *Harpullia pendula* | KM894421 | KM895007 | KM895496 | P.I.Forster PIF38510 |
| *Hedraianthera porphyropetala* | KM894729 |  | KM895874 | W.J. McDonald 2012-01-012 |
| *Hedycarya angustifolia* | KM894544 |  | KM895649 | P.I.Forster PIF38727 |
| *Helicia ferruginea* | KM894793 | KM895273 | KM895953 | G. Smyrell GS-32-2 |
| *Helicia glabriflora* |  |  | KM895778 | P.Dufourq GPP |
| *Hernandia bivalvis* | KM894574 | KM895117 | KM895684 | P.Grimshaw G134 |
| *Hibbertia scandens* |  | KM895326 | KM896036 | Smyrell;Kupsch |
| *Hibiscus heterophyllus* | KM894805 | KM895281 | KM895970 | ShapcottHiHe1-1 |
| *Hibiscus splendens* | KM894510 |  | KM895602 | G.Smyrell GS-28-1 |
| *Hibiscus tiliaceus* |  | KM895323 | KM896032 | ShapcottHiTi1-1 |
| *Hicksbeachia pinnatifolia* |  |  | KM895899 | W.J.McDonald 2012-01-010 |
| *Hippocratea barbata* | KM894624 | KM895156 | KM895738 | P.Dufourq NCCP |
| *Hodgkinsonia ovatiflora* | KM894950 | KM895395 | KM896151 | P.I.Forster PIF38722 |
| *Homalanthus nutans* |  |  | KM896071 | B.Jeffers |
| *Homalanthus stillingiifolius* |  |  | KM896113 | ShapcottHoSt1-1 |
| *Homalium alnifolium* | KM894800 | KM895278 | KM895964 | G.Smyrell GS-1-4 |
| *Hovea longipes* | KM894688 |  | KM895825 | P.I.Forster PIF38596 |
| *Hoya australis* | KM894655 |  | KM895781 | ShapcottHoAu1-1 |
| *Hydrocotyle pedicellosa* | KM894501 | KM895069 | KM895590 | ShapcottHyPe1-1 |
| *Hymenosporum flavum* | KM894958 | KM895398 | KM896163 | ShapcottHyFl1-1 |
| *Hypserpa decumbens* | KM894794 | KM895274 | KM895955 | G.Smyrell GS-32-4 |
| *Ixora beckleri* | KM894836 |  | KM896007 | PI Forster PI38974 |
| *Ixora queenslandica* | KM894707 | KM895216 |  | G.Smyrell GS-20-1 |
| *Jagera pseudorhus* |  | KM895285 | KM895976 | P.Dufourq BC |
| *Jasminum didymum* | KM894815 | KM895292 | KM895981 | ShapcottJaDi1-1 |
| *Jasminum jenniae* | KM894578 | KM895121 | KM895689 | Leiper AQ763154; ex cult |
| *Jasminum simplicifolium* | KM894814 | KM895289 | KM895979 | P.I.Forster PIF38570 |
| *Jasminum singuliflorum* | KM894571 | KM895113 | KM895678 | P.I.Forster PIF38356 |
| *Legnephora moorei* | KM894736 | KM895236 | KM895881 | P.Dufourq NCCP |
| *Leionema elatius* | KM894650 |  | KM895775 | P.I.Forster PIF38756 |
| *Lenwebbia prominens* | KM894619 | KM895153 | KM895733 | P.I.Forster PIF38712 |
| *Lepiderema punctulata* | KM894689 |  | KM895826 | G.Smyrell GS-10-10 |
| *Ligustrum australianum* |  | KM895017 | KM895515 | G. Smyrell GS-IN-13 |
| *Linospadix monostachya* | KM894668 |  | KM895799 | Shapcott LiMo1-1 |
| *Litsea australis* |  |  | KM895601 | P.Dufourq GPP |
| *Litsea fawcettiana* |  |  | KM895650 | G.Smyrell GS-12-3 |
| *Litsea leefeana* | KM894622 | KM895185 | KM895736 | ShapcottLiLe1-1 |
| *Litsea reticulata* | KM894425 | KM895204 | KM895502 | G.Smyrell GS-7-7 |
| *Livistona australis* | KM894923 |  | KM896109 | ShapcottLiAu1-1 |
| *Livistona decora* | KM894900 |  | KM896082 | G. Smyrell GS-IN-14 |
| *Lomandra confertifolia* | KM894549 |  | KM895655 | G.Smyrell GS-5-4 |
| *Lomandra hystrix* | KM894415 | KM895002 | KM895490 | R.Etherington NCCP |
| *Lomandra longifolia* | KM894981 | KM895416 | KM896186 | G.Smyrell GS-24-5 |
| *Lomandra spicata* |  |  | KM896089 | G.Smyrell GS-7-9 |
| *Lomatia arborescens* | KM894927 | KM895374 | KM896115 | P.I.Forster PIF38772 |
| *Lophostemon confertus* |  | KM895042 | KM895549 | ShapcottLoCo1-1 |
| *Lophostemon suaveolens* | KM894491 | KM895061 |  | G.Smyrell GS-12-2 |
| *Lysiphyllum carronii* |  | KM895381 | KM896133 | ShapcottLyCa1-1 |
| *Lysiphyllum hookeri* |  | KM895378 | KM896124 | ShapcottLyHo1-1 |
| *Macadamia integrifolia* | KM894964 |  | KM896169 | Guymer D1 |
| *Macadamia jansenii* |  |  | KM895739 | ShapcottMaJa1-1 |
| *Macadamia ternifolia* | KM894561 |  | KM895667 | ShapcottMaTer1-1 |
| *Macadamia tetraphylla* | KM894427 |  | KM895504 | Powell |
| *Macaranga tanarius* |  |  | KM896111 | Shapcott MaTa1-1 |
| *Mackinlaya macrosciadea* | KM894787 |  | KM895949 | G.Smyrell GS-31-1 |
| *Maclura cochinchinensis* | KM894681 |  |  | Thomas |
| *Macropteranthes leiocaulis* |  |  | KM895554 | G. Smyrell GS-IN-7 |
| *Mallotus claoxyloides* |  |  | KM895643 | P.Dufourq MFNP |
| *Mallotus ficifolius* |  |  | KM895612 | G. Smyrell GS-40-6 |
| *Mallotus megadontus* |  |  | KM895830 | shapcottMaMe1-1 |
| *Mallotus mollissimus* |  |  | KM895720 | G. Smyrell GS-40-1 |
| *Mallotus philippensis* |  | KM895188 | KM895792 | P.Dufourq MFNP |
| *Marsdenia hemiptera* | KM894416 | KM895003 | KM895491 | ShapcottMaHe1-1 |
| *Marsdenia lloydii* | KM894508 | KM895074 | KM895598 | PI Forster PIF38967 |
| *Marsdenia micradenia* | KM894572 | KM895115 | KM895681 | G.Smyrell GS-30-2 |
| *Marsdenia pleiadenia* | KM894790 |  | KM895951 | P.I.Forster PIF38593 |
| *Marsdenia rostrata* | KM894448 |  | KM895527 | G.Smyrell GS-10-1 |
| *Medicosma cunninghamii* | KM894701 |  | KM895843 | P.I.Forster PIF38600 |
| *Medicosma elliptica* |  | KM895403 | KM896174 | G. Smyrell GS-IN-8 |
| *Meiogyne stenopetala* | KM894528 | KM895085 | KM895626 | P.I.Forster PIF38346 |
| *Melaleuca bracteata* | KM894989 |  | KM896199 | L.Simmons |
| *Melaleuca styphelliodes* |  |  | KM895820 | G.Smyrell GS-30-1 |
| *Melia azedarach* |  |  | KM895647 | P.Dufourq BC |
| *Melicope elleryana* | KM894584 | KM895127 | KM895695 | ShapcottMeEl1-1 |
| *Melicope hayesii* |  |  | KM895537 | P.I.Forster PIF38724 |
| *Melicope micrococca* | KM894486 | KM895058 | KM895576 | Shapcott MeMi1-1 |
| *Melicope vitiflora* | KM894552 |  | KM895659 | G.Smyrell GS-7-8 |
| *Melodinus acutiflorus* |  |  |  | P.Dufourq NCCP |
| *Melodinus australis* | KM894698 |  | KM895840 | Shapcott MeAu1-1 |
| *Melodorum leichhardtii* |  | KM895052 | KM895567 | P.Dufourq GPP |
| *Memecylon pauciflorum* |  |  |  | G.Smyrell GS-15-2 |
| *Micromelum minutum* |  | KM895033 | KM895535 | G.Smyrell GS-10-9 |
| *Mimusops elengi* | KM894483 |  | KM895573 | Mathieson MTM1242 |
| *Mischarytera lautereriana* |  | KM895077 | KM895606 | P.Dufourq GPP |
| *Mischocarpus ailae* | KM894906 | KM895356 |  | C.Booth s.n. |
| *Mischocarpus anodontus* |  |  | KM895813 | G.Smyrell GS-2-6 |
| *Mischocarpus australis* | KM894926 | KM895203 | KM896114 | Halford QM560 |
| *Mischocarpus pyriformis* | KM894855 |  | KM896025 | P.I.Forster PIF38253 |
| *Monococcus echinophorus* |  | KM895303 | KM896003 | P.Dufourq NCCP |
| *Mucuna gigantea* | KM894634 | KM895166 | KM895753 | shapcottMuGi1-1 |
| *Muellerina myrtifolia* |  | KM895120 | KM895688 | P.I.Forster PIF38540 |
| *Murraya ovatifoliolata* |  | KM895365 | KM896103 | ShapcottMuOv1-1 |
| *Myoporum acuminatum* | KM894414 | KM895000 | KM895488 | SHapcottMyAc1-1 |
| *Myoporum betcheanum* | KM894997 |  | KM896210 | PIForster PIF38970 |
| *Myrsine howittiana* | KM894463 | KM895041 | KM895546 | Shaw SS1 |
| *Myrsine ireneae* |  |  | KM895930 | G. Smyrell GS-41-7 |
| *Myrsine porosa* | KM894607 |  |  | Mathieson MTM1280 |
| *Myrsine subsessilis* | KM894977 | KM895343 | KM896180 | P.I.Forster PIF38699 |
| *Myrsine variabilis* | KM894577 | KM895119 | KM895687 | P.I.Forster PIF38552 |
| *Nauclea orientalis* | KM894687 |  |  | G.Smyrell GS-40-3 |
| *Neoachmandra cunninghamii* | KM894500 | KM895068 |  | Thomas |
| *Neolitsea australiensis* | KM894545 |  | KM895651 | Shapcott NeAu1-1 |
| *Neolitsea brassii* |  | KM895422 | KM896201 | G. Smyrell GS-11-3 |
| *Neolitsea dealbata* | KM894823 |  | KM895990 | P.Dufourq DNP |
| *Niemeyera chartacea* | KM894744 |  | KM895896 | P.Dufourq GPP |
| *Niemeyera whitei* |  |  | KM896073 | W.J.McDonald 2012-01-011 |
| *Notelaea johnsonii* | KM894438 | KM895019 | KM895517 | G.Smyrell GS-6-6 |
| *Notelaea lloydii* | KM894617 | KM895151 | KM895729 | G. Smyrell GS-IN -4 |
| *Notelaea longifolia* | KM894494 | KM895064 | KM895584 | Smyrell;Kupsch |
| *Notelaea microcarpa* | KM894756 | KM895255 | KM895910 | G.Smyrell GS-1-5 |
| *Nothofagus moorei* |  |  | KM895644 | P.I.Forster PIF38705 |
| *Nyssanthes diffusa* |  |  | KM895941 | G. Smyrell GS-36-4 |
| *Ochrosia moorei* |  | KM895423 | KM896203 | P.I.Forster PIF38743 |
| *Oldenlandia gibsonii* | KM894589 |  |  | G. Smyrell GS-IN-25 |
| *Olea paniculata* |  |  | KM895529 | P.Dufourq MFNP |
| *Olearia canescens* | KM894829 |  | KM895998 | P.I.Forster PIF38594 |
| *Olearia elliptica* | KM894733 | KM895235 | KM895878 | P.I.Forster PIF38542 |
| *Orites excelsus* | KM894663 |  | KM895794 | P.I.Forster PIF38688 |
| *Owenia acidula* | KM894980 |  | KM896185 | BJ8 |
| *Owenia venosa* | KM894658 | KM895332 | KM895786 | P.I.Forster PIF38677 |
| *Ozothamnus bidwillii* | KM894469 | KM895049 | KM895559 | P.I.Forster PIF38564 |
| *Ozothamnus rufescens* | KM894749 | KM895249 | KM895902 | P.I.Forster PIF 38969 |
| *Palmeria foremanii* | KM894534 |  |  | W.J.McDonald 2012-02-011 |
| *Pandorea baileyana* | KM894810 | KM895284 | KM895975 | P.I.Forster PIF38725 |
| *Pandorea floribunda* | KM894612 | KM895144 | KM895724 | B. Jeffers |
| *Pandorea jasminoides* | KM894818 |  | KM895983 | L. Simmons |
| *Pararchidendron pruinosum* | KM894880 |  | KM896057 |  |
| *Pararistolochia laheyana* | KM894435 | KM895016 | KM895514 | W.J.McDonald 2012-02-002 |
| *Pararistolochia praevenosa* | KM894852 |  |  | P.I.Forster PIF38363 |
| *Parsonsia eucalyptophylla* | KM894774 |  | KM895933 | P.I.Forster PIF38588 |
| *Parsonsia fulva* | KM894473 |  | KM895562 | G.Smyrell GS-37-1 |
| *Parsonsia lanceolata* | KM894834 | KM895307 | KM896006 | P.I.Forster PIF38678 |
| *Parsonsia largiflorens* | KM894745 |  | KM895897 | G.Smyrell GS-7-10 |
| *Parsonsia leichhardtii* | KM894847 | KM895314 | KM896018 | P.I.Forster PIF29453 |
| *Parsonsia lilacina* | KM894615 | KM895148 |  | G.Smyrell GS-7-11 |
| *Parsonsia longipetiolata* | KM894553 | KM895103 |  | P.I.Forster PIF 38563 |
| *Parsonsia paulforsteri* | KM894639 | KM895168 | KM895759 | G. Smyrell GS-53-5 |
| *Parsonsia plaesiophylla* | KM894488 |  | KM895578 | G.Smyrell GS-18-1 |
| *Parsonsia rotata* | KM894870 | KM895330 | KM896043 | G.Smyrell GS-13-2 |
| *Parsonsia sankowskyana* | KM894872 | KM895333 |  | G.Smyrell GS-18-2 |
| *Parsonsia straminea* | KM894808 |  | KM895973 | Guymer D33 |
| *Parsonsia tenuis* | KM894600 | KM895135 | KM895709 | P.I.Forster PIF38773 |
| *Parsonsia velutina* | KM894675 | KM895196 | KM895809 | G. Smyrell GS-33-1 |
| *Parsonsia ventricosa* | KM894616 | KM895150 | KM895728 | P.I.Forster PIF38362 |
| *Passiflora aurantia* |  | KM895202 | KM895824 | P.Dufourq BMNP |
| *Passiflora herbertiana* | KM894789 |  | KM895950 | G. Smyrell GS-48-2 |
| *Pavetta australiensis* |  | KM895399 | KM896165 | Mathieson MTM1243 |
| *Pennantia cunninghamii* | KM894816 | KM895293 | KM895982 | P.I.Forster PIF38558 |
| *Pentaceras australe* | KM894764 |  | KM895918 | G.Smyrell GS-36-2 |
| *Peperomia blanda* |  |  | KM895679 | P.I.Forster PIF39008 |
| *Persoonia amaliae* | KM894555 |  | KM895661 | Young |
| *Persoonia media* | KM894502 | KM895070 |  | P.I.Forster PIF38729 |
| *Petalostigma pubescens* |  | KM895389 |  | Smyrell;Kupsch |
| *Petalostigma triloculare* | KM894874 | KM895336 | KM896051 | Smyrell;Kupsch |
| *Petermannia cirrosa* |  | KM895106 | KM895668 | W.J.McDonald 2012-01-006 |
| *Phaleria chermsideana* | KM894762 | KM895257 | KM895917 | P.I.Forster PIF38544 |
| *Phebalium distans* | KM894947 | KM895388 | KM896144 | G. Smyrell GS-5--1 |
| *Phyllanthus gunnii* | KM894461 | KM895038 | KM895543 | P.I.Forster PIF39013 |
| *Phyllanthus microcladus* | KM894957 |  | KM896161 | P.Dufourq KFNPlb |
| *Phyllanthus sauropodoides* | KM894759 |  | KM895914 | G.Smyrell GS-40-2 |
| *Phyllanthus sp. Bulburin* | KM894937 |  | KM896130 | G. Smyrell GS -41-8 |
| *Phyllanthus subcrenulatus* | KM894807 |  | KM895972 | P.I.Forster PIF38730 |
| *Pilidiostigma glabrum* | KM894832 | KM895305 | KM896004 | W.J.McDonald 2012-02-004 |
| *Pilidiostigma rhytispermum* |  |  | KM896044 | P.Dufourq MFNP |
| *Pimelea latifolia* | KM894735 |  | KM895880 | BJ20 |
| *Pimelea ligustrina* | KM894656 | KM895182 | KM895784 | P.I.Forster PIF25384 |
| *Pimelea neoanglica* | KM894853 |  | KM896023 | BJ16 |
| *Piper hederaceum* |  | KM895001 | KM895489 | Smyrell;Kupsch |
| *Pipturus argenteus* | KM894693 |  |  | P.Dufourq KFNP |
| *Pittosporum angustifolium* | KM894430 | KM895012 | KM895508 | P.I.Forster PIF39007 |
| *Pittosporum ferrugineum* | KM894548 |  | KM895654 | ShapcottPiFe1-1 |
| *Pittosporum lancifolium* | KM894564 |  | KM895670 | W.J.McDonald 2012-02-001 |
| *Pittosporum multiflorum* |  | KM895370 | KM896108 | Halford QM562 |
| *Pittosporum oreillyanum* | KM894657 | KM895183 | KM895785 | P.I.Forster PIF38709 |
| *Pittosporum revolutum* | KM894960 |  |  | P.Dufourq MFNP |
| *Pittosporum spinescens* | KM894692 | KM895206 | KM895829 | ShapcottPiSp1-1 |
| *Pittosporum undulatum* | KM894821 | KM895297 | KM895988 | P.Dufourq KFNP |
| *Pittosporum viscidum* | KM894518 |  | KM895614 | G.Smyrell GS-3-6 |
| *Planchonella australis* | KM894587 | KM895116 |  | P.I.Forster PIF38392 |
| *Planchonella chartacea* | KM894891 |  | KM896068 | P.I.Forster PIF38507 |
| *Planchonella cotinifolia* | KM894432 | KM895014 | KM895511 | ShapcottPlCo1-1 |
| *Planchonella eerwah* | KM894796 |  | KM895956 | P.I.Forster PIF38776 |
| *Planchonella myrsinifolia* | KM894894 |  | KM896072 | P.I.Forster PIF38300 |
| *Planchonella pohlmaniana* | KM894424 |  | KM895501 | G.Smyrell GS-9-2 |
| *Planchonia careya* | KM894763 |  |  | G.Smyrell GS-9-3 |
| *Pleiogynium timorense* | KM894951 | KM895348 | KM896152 | ShapcottPlTi1-1 |
| *Pleogyne australis* | KM894994 |  | KM896206 | G Smyrell GS-24-4 |
| *Pleurostylia opposita* | KM894417 | KM895004 | KM895492 | G.Smyrell GS-30-3 |
| *Podocarpus elatus* |  |  | KM896187 | P.Dufourq MFNP |
| *Pollia crispata* |  |  | KM895594 | P.Dufourq MFNP |
| *Pollia macrophylla* |  |  | KM895510 | P.I.Forster PIF38522 |
| *Polyalthia nitidissima* | KM894526 | KM895083 | KM895624 | Young |
| *Polyosma cunninghamii* |  |  | KM895927 | G.Smyrell GS-7-12 |
| *Polyscias elegans* | KM894550 | KM895101 | KM895657 | ShapcottPoEl1-1 |
| *Polyscias murrayi* | KM894838 | KM895310 | KM896010 | ShapcottPoMu1-1 |
| *Polyscias sambucifolia* | KM894876 |  | KM896053 | P.I.Forster PIF38832 |
| *Pomaderris clivicola* |  |  | AJ390063.1 | G. Smyrell GS-IN-17 |
| *Pothos longipes* | KM894458 | KM895037 | KM895540 | P.Dufourq GPP |
| *Pouteria queenslandica* | KM894671 | KM895371 | KM895802 | G.Smyrell GS-6-7 |
| *Pseuderanthemum tenellum* |  | KM895124 | KM895692 | G.Smyrell GS-41-3 |
| *Pseudoweinmannia lachnocarpa* | |  | KM896192 | ShapcottPSLa1-1 |
| *Psychotria daphnoides* |  |  | KM895682 | ShapcottPsDa1-1 |
| *Psychotria loniceroides* | KM894504 |  | KM895592 | P.Duforq MFNP |
| *Psychotria simmondsiana* |  | KM895376 | KM896118 | P.I.Forster PIF38883 |
| *Psydrax lamprophylla* | KM894922 | KM895368 |  | ShapcottPsLa1-1 |
| *Psydrax odorata* | KM894884 | KM895342 | KM896060 | G. Smyrell GS-49-12 |
| *Quintinia sieberi* |  | KM895210 | KM895837 | P.I.Forster PIF38740 |
| *Quintinia verdonii* | KM894990 | KM895267 | KM896202 | P.I.Forster PIF38719 |
| *Rhagodia spinescens* | KM894897 |  | KM896078 | B. Jeffers |
| *Rhamnella vitiensis* | KM894562 |  |  | G.Smyrell GS-10-11 |
| *Rhodamnia acuminata* | KM894659 | KM895184 | KM895787 | ShapcottRhAc1-1 |
| *Rhodamnia angustifolia* | KM894678 | KM895199 |  | G. Smyrell GS-IN-10 |
| *Rhodamnia argentea* | KM894835 | KM895308 |  | P.I.Forster PIF38342 |
| *Rhodamnia dumicola* | KM894465 | KM895043 | KM895550 | G.Smyrell GS-31-2 |
| *Rhodamnia glabrescens* | KM894954 |  | KM896156 | G. Smyrell GS-41-11 |
| *Rhodamnia maideniana* | KM894813 | KM895288 | KM895978 | P.I.Forster PIF38839 |
| *Rhodamnia pauciovulata* | KM894974 | KM895408 |  | Guymer D7 |
| *Rhodamnia rubescens* | KM894700 | KM895213 | KM895842 | Guymer D12 |
| *Rhodamnia whiteana* | KM894670 | KM895192 | KM895801 | PI Forster PIF38964 |
| *Rhodomyrtus psidioides* |  | KM895187 | KM895791 | G.Smyrell GS-31-5 |
| *Rhodosphaera rhodanthema* | KM894755 |  | KM895909 | P.I.Forster PIF38993 |
| *Rhysotoechia bifoliolata* | KM894911 | KM895363 |  | Shapcott RhBi1-1 |
| *Ricinocarpos ledifolius* |  |  | KM895566 | G. Smyrell GS-IN-9 |
| *Ricinocarpos speciosus* | KM894556 |  | KM895662 | P.I.Forster PIF38742 |
| *Ripogonum album* | KM894450 | KM895030 | KM895530 | P.I.Forster PIF38357 |
| *Ripogonum brevifolium* | KM894864 |  |  | P.I.Forster PIF38650 |
| *Ripogonum discolor* | KM894482 |  | KM895572 | P.I.Forster PIF38737 |
| *Ripogonum elseyanum* | KM894863 | KM895325 |  | P.Dufourq GPP |
| *Ripogonum fawcettianum* | KM894883 | KM895341 |  | P.I.Forster PIF38706 |
| *Romnalda strobilacea* | KM894961 | KM895400 | KM896166 | ShapcottRoSt1-1 |
| *Rubus moluccanus* | KM894798 |  | KM895961 | G.Smyrell GS-48-1 |
| *Rubus moorei* |  |  | KM896001 | PI Forster PIF38749 |
| *Rubus nebulosus* | KM894898 |  | KM896080 | P.I.Forster PIF38843 |
| *Rubus probus* | KM894901 | KM895352 | KM896083 | G. Smyrell GS-41-9 |
| *Rubus rosifolius* |  | KM895211 | KM895838 | P.Dufourq MMSF |
| *Samadera bidwillii* |  |  | KM895962 | G.Smyrell GS-6-8 |
| *Samadera sp. Mt Nardi* |  |  | KM896157 | Jinks |
| *Sambucus australasica* |  | KM895081 | KM895620 | P.I.Forster PIF38718 |
| *Santalum lanceolatum* | KM894652 |  | KM895777 | BJ 1 |
| *Sarcomelicope simplicifolia* | KM894966 |  | KM896170 | PI Forster PIF 38991 |
| *Sarcopetalum harveyanum* | KM894563 | KM895107 | KM895669 | P.I.Forster PIF38845 |
| *Sarcopteryx stipata* |  | KM895152 | KM895731 | P.Dufourq GPP |
| *Sarcostemma viminale* | KM894686 |  |  | Guymer D31 |
| *Sauropus albiflorus* | KM894539 |  | KM895640 | P.I.Forster PIF38781 |
| *Schefflera actinophylla* | KM894791 |  |  | ShapcottScAc1-1 |
| *Schizomeria ovata* | KM894531 | KM895087 | KM895629 | P.Dufourq MMSF |
| *Scolopia braunii* |  | KM895251 | KM895904 | ShapcottScBr1-1 |
| *Secamone elliptica* | KM894676 | KM895197 | KM895810 | G.Smyrell GS-3-7 |
| *Senecio amygdalifolius* | KM894753 |  | KM895906 | ShapcottSeAm1-1 |
| *Senna acclinis* | KM894877 |  | KM896054 | P.I.Forster PIF38873 |
| *Senna coronilloides* | KM894731 | KM895234 | KM895876 | BJ5 |
| *Senna sophera* |  | KM895080 | KM895619 | Guymer D16 |
| *Senna surattensis* |  | KM895269 | KM895940 | ShapcottSeSu1-1 |
| *Sersalisia sericea* |  |  | KF496488.1 | G.Smyrell GS-8-1 |
| *Sicyos australis* |  | KM895384 | KM896138 | ShapcottSiAu1-1 |
| *Siphonodon australis* | KM894704 | KM895214 |  | P.I.Forster PIF38541 |
| *Sloanea australis* | KM894713 | KM895218 |  | P.Dufourq GPP |
| *Sloanea woollsii* | KM894828 | KM895300 | KM895994 | ShapcottSlWo1-1 |
| *Smilax australis* | KM894846 |  | KM896017 | P.Dufourq MFNP |
| *Smilax glyciphylla* | KM894718 |  | KM895858 | P.Dufourq MFNP |
| *Solanum aviculare* | KM894419 | KM895005 | KM895494 | P.Dufourq BMNP |
| *Solanum callium* | KM894437 |  |  | ShapcottSoCa1-1 |
| *Solanum corifolium* | KM894596 |  | KM895705 | ShapcottSoCo1-1 |
| *Solanum densevestitum* | KM894858 | KM895321 | KM896028 | Guymer D29 |
| *Solanum mentiens* | KM894761 |  | KM895916 | PI Forster PIF28047 |
| *Solanum mitchellianum* | KM894460 |  | KM895542 | BJ17 |
| *Solanum nobile* | KM894748 |  | KM895901 | PI Forster PIF38965 |
| *Solanum parvifolium* | KM894603 |  | KM895712 | BJ21 |
| *Solanum semiarmatum* | KM894998 | KM895426 | KM896211 | L.Simmons |
| *Solanum shirleyanum* | KM894633 | KM895165 | KM895752 | P.Dufourq GPP |
| *Solanum stelligerum* | KM894464 |  | KM895547 | Guymer D28 |
| *Solanum vicinum* | KM894567 |  |  | P.I.Forster PIF38562 |
| *Spartothamnella juncea* | KM894784 | KM895271 | KM895946 | P.I.Forster PIF38599 |
| *Stenocarpus salignus* | KM894423 |  | KM895500 | P.I.Forster PIF38695 |
| *Stenocarpus sinuatus* | KM894674 |  | KM895806 | ShapcottStSi1-1 |
| *Stephania japonica* | KM894738 | KM895240 | KM895886 | G.Smyrell GS-19-3 |
| *Sterculia quadrifida* |  |  | KM896132 | ShapcottStQu1-1 |
| *Streblus pendulinus* | KM894939 |  | KM896134 | Shapcott StBr1-1 |
| *Streptothamnus moorei* | KM894503 | KM895071 | KM895591 | G. Leiper & L.Weber s.n. |
| *Strychnos psilosperma* | KM894777 |  | KM895936 | G. Smyrell GS-49-2 |
| *Symplocos baeuerlenii* | KM894627 | KM895158 | KM895743 | P.I.Forster PIF38735 |
| *Symplocos harroldii* | KM894638 |  | KM895758 | G.Smyrell 39-1 |
| *Symplocos stawellii* | KM894819 | KM895295 | KM895986 | G. Smyrell GS-44-2 |
| *Syncarpia glomulifera* | KM894993 |  |  | Shapcott SyGl1-1 |
| *Syncarpia hillii* |  |  | KM895675 | L.Simmons |
| *Synoum glandulosum* | KM894804 | KM895280 | KM895969 | ShapcottSyGl1-1 |
| *Syzygium australe* |  | KM895253 | KM895907 | P.Dufourq NCCP |
| *Syzygium corynanthum* |  |  | KM896194 | P.I.Forster PIF38875 |
| *Syzygium crebrinerve* |  |  |  | P.I.Forster PIF38361 |
| *Syzygium francisii* | KM894913 | KM895364 | KM896098 | Thomas |
| *Syzygium hodgkinsoniae* |  | KM895409 | KM896178 | P.Dufourq GPP |
| *Syzygium luehmannii* | KM894967 | KM895401 | KM896171 | P.I.Forster PIF38508 |
| *Syzygium oleosum* | KM894519 |  | KM895615 | ShapcottSzOl1-1 |
| *Tabernaemontana pandacaqui* | KM894585 |  | KM895696 | P.Dufourq GPP |
| *Tapeinosperma repandulum* | KM894724 | KM895227 | KM895867 | P.Dufourq GPP |
| *Tasmannia insipida* |  |  | KM895891 | P.Dufourq KFNPlb |
| *Tecomanthe hillii* | KM894740 | KM895242 | KM895888 | L.Simmons |
| *Terminalia porphyrocarpa* | KM894936 |  |  | ShapcottTePo1-1 |
| *Tetrastigma nitens* | KM894882 | KM895339 | KM896059 | Guymer D32 |
| *Teucrium sp. Ormeau* | KM894929 |  | KM896119 | AQ541446 |
| *Timonius timon* | KM894726 | KM895229 | KM895870 | Mathieson MTM1234 |
| *Tinospora smilacina* | KM894831 | KM895302 | KM896002 | G.Smyrell GS-10-6 |
| *Tinospora tinosporoides* | KM894867 | KM895328 | KM896038 | PI Forster PIF28795 |
| *Toechima dasyrrhache* |  | KM895173 | KM895765 | G. Smyrell GS-44-6 |
| *Toechima tenax* | KM894568 | KM895110 | KM895676 | P.I.Forster PIF38778 |
| *Toona ciliata* |  | KM895079 | KM895616 | P.Dufourq BMNP |
| *Tragia novae-hollandiae* |  |  | KM895797 | G.Smyrell GS-9-4 |
| *Trema orientalis* | KM894431 | KM895013 | KM895509 | G. Smyrell GS-46-2 |
| *Trema tomentosa* | KM894497 | KM895066 | KM895587 | P.Dufourq BC |
| *Trichosanthes subvelutina* | KM894965 |  |  | G. Smyrell GS-48-7 |
| *Triflorensia cameronii* | KM894684 |  | KM895819 | P.I.Forster PIF38686 |
| *Triflorensia ixoroides* | KM894527 | KM895084 | KM895625 | G. Smyrell GS-49-7 |
| *Trimenia moorei* | KM894599 | KM895134 | KM895708 | G. Leiper & L.Weber s.n. |
| *Tristaniopsis collina* | KM894817 |  |  | P.I.Forster PIF38728 |
| *Tristaniopsis laurina* | KM894782 |  | KM895945 | Guymer D35 |
| *Triunia robusta* | KM894540 |  | KM895641 | ShapcottTrRo1-1 |
| *Triunia youngiana* | KM894809 | KM895283 | KM895974 | ShapcottTrYo1-1 |
| *Trochocarpa laurina* | KM894428 | KM895010 | KM895506 | P.I.Forster PIF38759 |
| *Trophis scandens* |  | KM895143 | KM895723 | ShapcottTrSc1-1 |
| *Turraea pubescens* | KM894776 |  | KM895935 | G.Smyrell GS-2-8 |
| *Tylophora benthamii* | KM894479 | KM895054 |  | P.I.Forster PIF38526 |
| *Tylophora paniculata* | KM894506 |  | KM895596 | P.Dufourq MMSF |
| *Uromyrtus lamingtonensis* | KM894902 | KM895354 | KM896085 | G. Leiper AQ636259 |
| *Urtica incisa* |  | KM895176 | KM895773 | ShapcottUrIn1-1 |
| *Ventilago ecorollata* |  |  | KM895639 | Guymer D9 |
| *Ventilago pubiflora* |  | KM895335 | KM896050 | G.Smyrell GS-15-1 |
| *Vitex lignum-vitae* | KM894602 |  |  | G.Smyrell GS-29-4 |
| *Vitex melicopea* | KM894988 | KM895421 | KM896198 | G.Smyrell GS-30-4 |
| *Waterhousea floribunda* | KM894811 |  |  | P.Dufourq KFNPlb |
| *Wikstroemia indica* | KM894856 | KM895318 | KM896026 | P.Dufourq MFNP |
| *Wilkiea austroqueenslandica* | KM894611 | KM895142 | KM895722 | P.I.Forster PIF38343 |
| *Wilkiea huegeliana* | KM894910 |  | KM896096 | P.I.Forster PIF38693 |
| *Wilkiea macrophylla* | KM894559 | KM895060 | KM895665 | P.Dufourq KFNP |
| *Xanthostemon oppositifolius* |  |  | KM896125 | G.Smyrell GS-30-10 |
| *Xylocarpus granatum* | KM894474 | KM895050 | KM895563 | G.Smyrell GS-29-6 |
| *Xylosma terrae-reginae* | KM894887 |  | KM896063 | G. Smyrell GS-IN-6 |
| *Zanthoxylum brachyacanthum* | KM894443 | KM895024 | KM895523 | P.I.Forster PIF38692 |
| *Zieria collina* | KM894851 | KM895316 | KM896022 | Halford Q7044A; ex cult |
| *Zieria smithii* | KM894941 | KM895382 | KM896136 | P.I. Forster PIF38461 |
| *Zieria vagans* | KM894444 | KM895026 |  | P.I.Forster PIF34832; ex cult |
| *Zieria verrucosa* | KM894973 | KM895407 | KM896177 | L.Simmons |
